# Total synthesis of lindbladione, a Hes1 dimerization inhibitor and neural stem cell activator isolated from *Lindbladia tubulina*

**DOI:** 10.1038/s41598-020-78524-7

**Published:** 2020-12-08

**Authors:** Midori A. Arai, Yuna Makita, Yumi Yamaguchi, Haruka Kawano, Akiko Suganami, Yutaka Tamura, Masami Ishibashi

**Affiliations:** 1 Department of Biosciences and Informatics, Faculty of Science and Technology, Keio University, 3-14-1 Hiyoshi, Kohoku-ku, Yokohama 223-8522 Japan; 2 Graduate School of Pharmaceutical Sciences, Chiba University, 1-8-1 Inohana, Chuo-ku, Chiba 260-8675 Japan; 3 Graduate School of Medicine, Chiba University, 1-8-1 Inohana, Chuo-ku, Chiba 260-8670 Japan

**Keywords:** Natural product synthesis, Chemistry, Chemical biology, Natural products, Natural product synthesis, Chemical biology, Natural products

## Abstract

Lindbladione (**1**) is a neural stem cell differentiation activator isolated from *Lindbladia tubulina* by our group. Hes1 dimerization inhibitory activity of lindbladione (**1**) was discovered using our original fluorescent Hes1 dimer microplate assay. We also found that lindbladione (**1**) accelerates the differentiation of neural stem cells. We conducted the first total synthesis of lindbladione (**1**) via Heck reaction of 1-hexene-3-one **7** with iodinated naphthoquinone **12**, which was provided by Friedel–Crafts acylation followed by Claisen condensation, in the presence of Pd (II) acetate. Careful deprotection of the benzyl groups of **13** successively provided lindbladione (**1**). Synthesized lindbladione (**1**) exhibited potent Hes1 dimer inhibition (IC_50_ of 2.7 μM) in our previously developed fluorescent Hes1 dimer microplate assay. Synthesized lindbladione (**1**) also accelerated the differentiation of C17.2 mouse neural stem cells into neurons dose dependently, increasing the number of neurons by 59% (2.5 μM) and 112% (10 μM) compared to the control. These activities are comparable to those of naturally occurring lindbladione (**1**) isolated from *L. tublina*.

## Introduction

Neurodegenerative diseases, such as Alzheimer's disease and Parkinson's disease, affect many body activities, such as balance, movement, and talking. Millions of people worldwide are affected by these diseases, which severely compromise their quality of life. The discovery of neural stem cells (NSCs) in human adult brain^1,2^ led to an expectation of novel treatments involving activation of NSC differentiation in the brain and NSC transplantation to diseased tissues. NSCs are self-renewing, multipotent cells that provide new neural cells such as neurons, astrocytes, and oligodendrocytes. Acceleration of NSC differentiation in the brain is an attractive approach to overcome neurodegenerative diseases. However, the development of regenerative medicine and small-molecule NSC activators has been slow due to the complex mechanism underlying the differentiation of NSCs into neural cells. In addition, internal NSCs are reportedly quiescent^3^.

The fate of NSCs, whether self-renewal or differentiation into neural cells, is controlled by basic helix-loop-helix (bHLH) transcriptional factors^4^. Proneural bHLH factors, such as Ascl1 (formerly Mash1) and Neurog2, accelerate neurogenesis. Hairy and enhancer of split (hes) factors, such as Hes1, promote the self-renewal of NSCs and the generation of astrocytes, and they also suppress the expression of proneural genes. Recently, Kageyama et al*.* reported that oscillations in the concentration of bHLH factors directly control the fate of NSCs^5–7^. Hes1 and Ascl1 proteins oscillate with 2- to 3-h periodicity in active NSCs to activate cell proliferation. During cell fate selection, one bHLH factor is expressed in a sustained manner while expression of the others is repressed. In neurons, Ascl1 is expressed sustainably, whereas Hes1 expression is inhibited. However, in the adult brain, NSCs are in quiescent state, with high expression of Hes1. Inactivation of Hes1 and up-regulation of Ascl1 expression leads to an increase in neurogenesis^7^.

**Figure 1:**
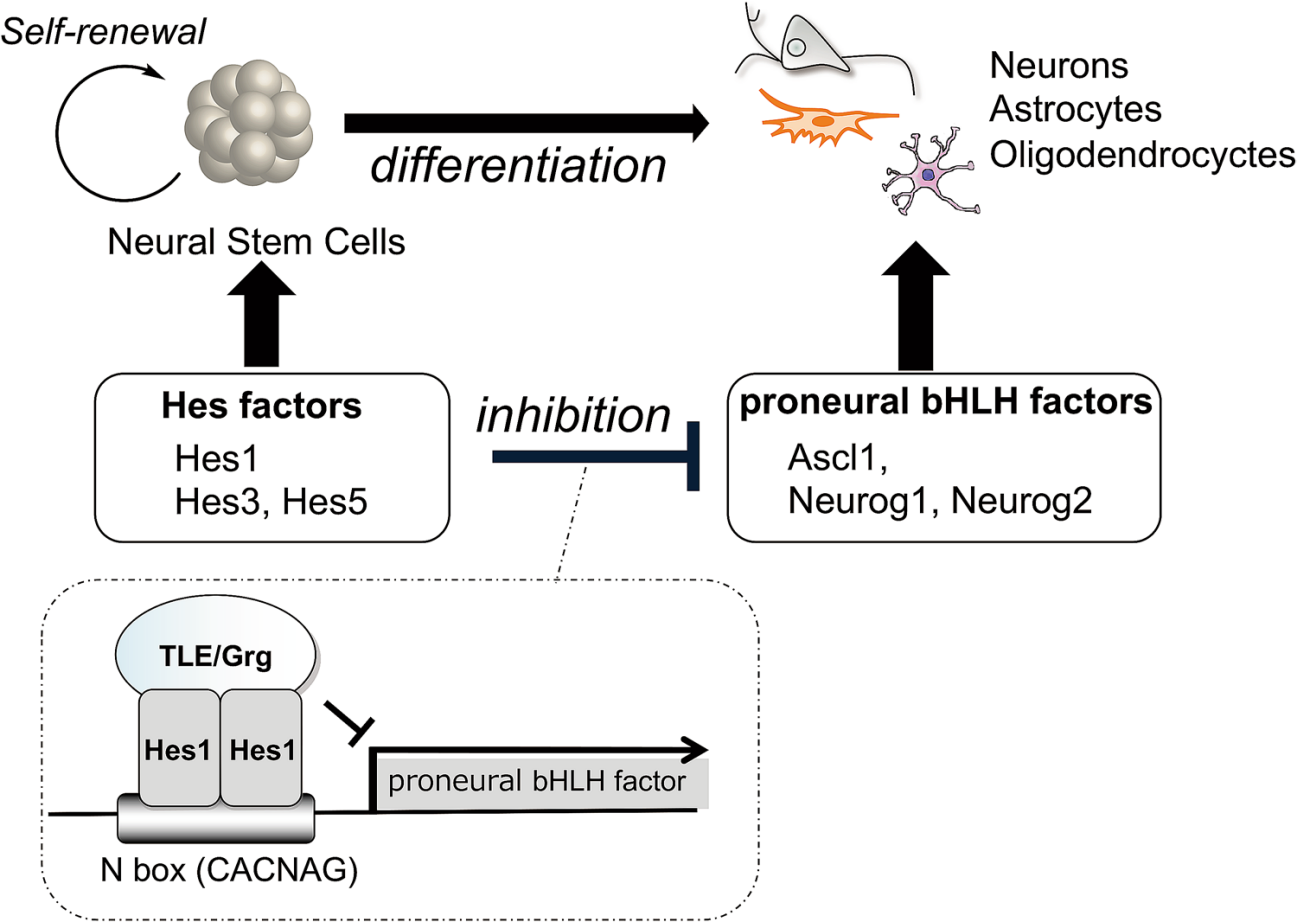
Neural stem cell differentiation and fate control by bHLH factors.

**Figure 2:**
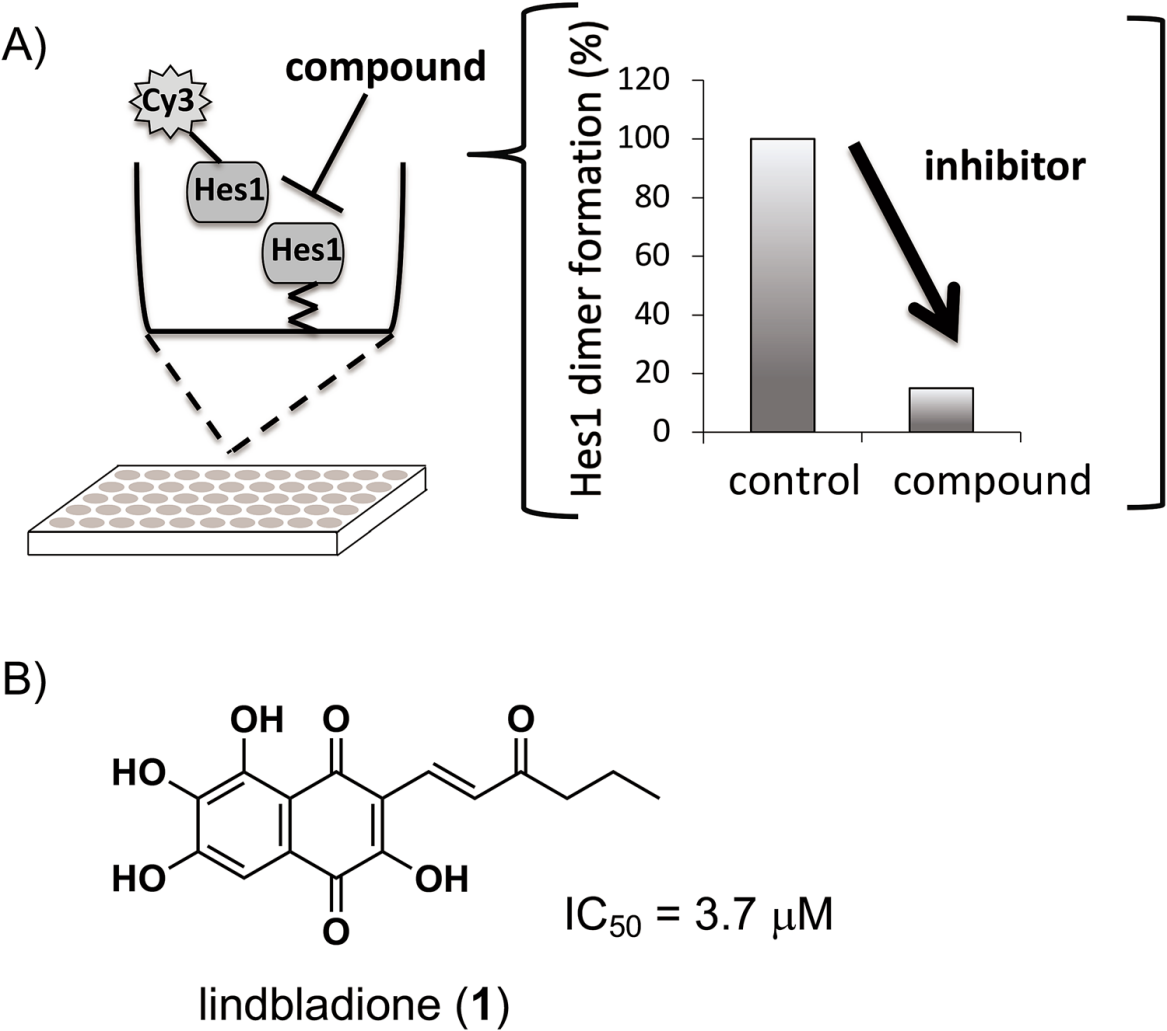
Screening of Hes1 dimerization inhibitors. (**A**) Hes1–Hes1 interaction fluorescent plate assay; (**B**) the first Hes1 dimerization inhibitor, lindbladione (**1**) and IC_50_ value of Hes1 dimer inhibition of natural product **1** (ref 14).

Because the Hes1 homodimer binds to the promoter region of *Ascl1* and other proneural genes to inhibit their expression (Fig. 1), the inhibition of Hes1 would be an attractive approach for enhancing neuronal differentiation. Inhibition of undesired protein–protein interactions (PPIs) is essential in drug development. Therefore, bioactivity-guided isolation of natural products using PPI inhibition is an attractive method for obtaining effective inhibitors. However, only a few examples of such approaches using PPI assay systems have been reported^8–12^. In our attempts to identify Hes1 inhibitors from extracts of natural resources composed of mixtures of natural products, we constructed a new Hes1–Hes1 interaction fluorescent plate assay, which yielded the first Hes1 dimerization inhibitors, including lindbladione (Fig. 2)^8^. Lindbladione increased the number of neurons via acceleration of NSC differentiation. We also developed “target protein oriented natural products isolation (TPO-NAPI) methods” using protein-immobilized beads^12–16^. Using Hes1 protein beads, agalloside, isomicromonolactam, inohanamine, and 4-*O*-(4″-*O*-galloyl-α-L-rhamnopyranosyl)ellagic acid were isolated as accelerators of the differentiation of NSCs into neurons^11,14,15^. Here, we report the first total synthesis of the Hes1 dimerization inhibitor lindbladione isolated from *Lindbladia tubulina*^17^. The attempted synthesis of lindbladione derivatives using a calculation approach is also reported.

## Results and discussion

**Figure 3:**
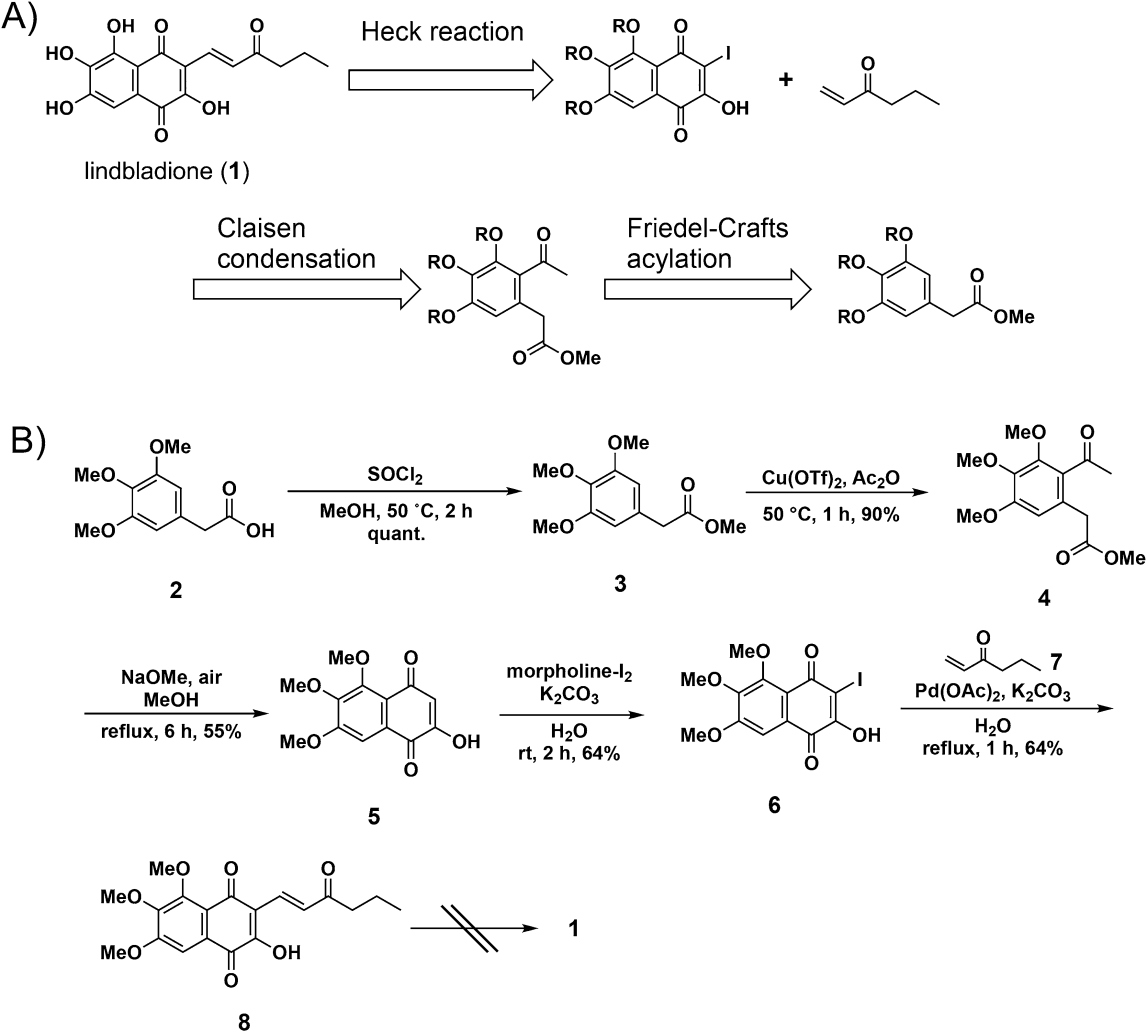
First trial of synthesis of **1**. (**A**) Retrosynthetic analysis of **1**. (**B**) Deprotection was a critical problem.

The results of retrosynthetic analyses are shown in Fig. 3A. Lindbladione (**1**) was considered to be obtained by the Heck reaction between 2-hydroxy-3-iodo naphthoquinone and α,β-unsaturated ketone, as in a previous report of naphthoquinone synthesis^18^. 2-Hydroxy-3-iodo naphthoquinone would be synthesized by Claisen condensation of the aromatic keto ester, followed by iodination. Friedel–Crafts acylation was selected to obtain the aromatic keto ester. Starting with trimethoxyphenylacetic acid **2**, chlorination by thionyl chloride followed by addition of MeOH gave methyl ester **3** (Fig. 3B). Friedel–Crafts acylation of **3** with acetic anhydride for 1 h proceeded smoothly to give keto ester **4** at 90% yield. Extending the reaction (3 h) resulted in a decrease in yield (51%). Claisen condensation of keto ester **4** in the presence of sodium methoxide gave cyclized product **5** at moderate yield. 2-Hydroxy-3-iodo naphthoquinone **6** was obtained by iodination of **5** by the morpholine-I_2_ complex after 2 h^19^. The Heck reaction of **6** with 1-hexene-3-one **7** and 10 mol% Pd(OAc)_2_ in the presence of 5 eq. of K_2_CO_3_ gave the desired product **8** at 64% yield. Although deprotection of the methyl ether was examined under several conditions (BBr_3_, TMSI, PhSH, AlCl_3_), all conditions failed to give lindbladione (**1**). Therefore, several protective groups were examined, including benzyl, acetyl, and methoxymethyl groups. However, Friedel–Crafts acylation did not proceed in all cases. Based on these results, the protective group was changed after the Friedel–Crafts acetylation.

**Figure 4:**
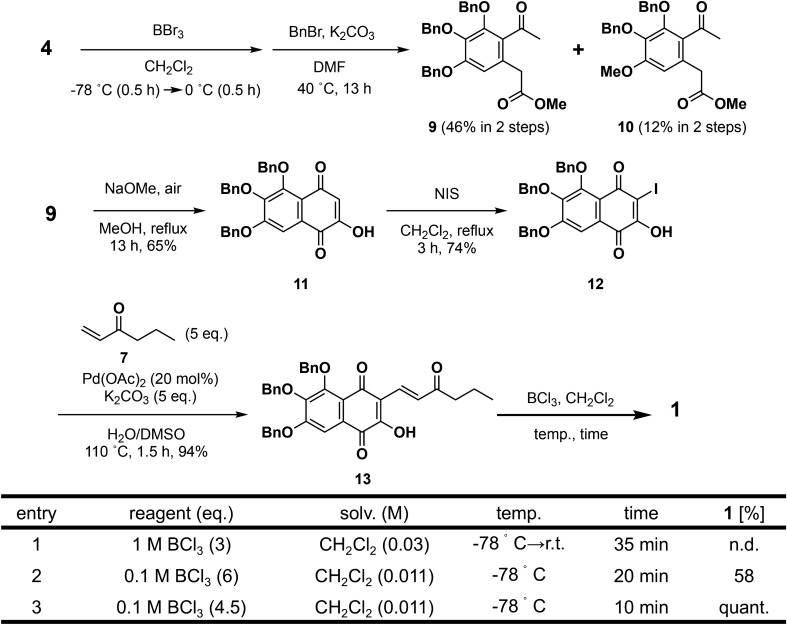
First total synthesis of **1**.

The deprotection of keto ester **4** by 3 eq. of BBr_3_ gave a mixture of trihydroxy- and dihydroxyphenyl compounds at a 4:1 ratio (Fig. 4). This mixture was treated with benzyl bromide in the presence of K_2_CO_3_ to give tribenzyl compound **9** and dibenzyl compound **10** at 46% and 12% yield, respectively. Electron withdrawing effects of the acetyl group decreased the electron density of the *para*-methoxy group, resulting in the escape of coordination by BBr_3_ and deprotection. Claisen condensation of **9** with sodium methoxide under air proceeded smoothly to give naphthoquinone **11** at 65% yield. Although the morpholine-I_2_ complex gave iodinated naphthoquinone **12** at low yield (33%), *N*-iodosuccinimide and CH_2_Cl_2_ reflux conditions gave **12** at 74% yield. The Heck reaction of **12** with 1-hexene-3-one **7** and 20 mol% Pd(OAc)_2_ in H_2_O-DMSO at 110 °C gave the benzyl-protected lindbladione **13**.

Next, we investigated deprotection of the benzyl group. Many conditions (Pd/H_2_, BBr_3_, trifluoroacetic acid, AlCl_3_ and NaI, BF_3_ and Me_2_S, TiCl_4_) resulted in failure to obtain the deprotected compound. However, careful addition of BCl_3_ (0.1 M, 4.5 eq.) to **13** in 0.011 M CH_2_Cl_2_ at − 78 °C (low-concentration condition) and a short reaction time (10 min) gave the target product lindbladione (**1**) at quantitative yield. Extending the reaction to 20 min decreased the yield (58%) due to the instability of **1** under these conditions. Data for NMR spectra (^1^H and ^13^C) and high-resolution mass spectra were all identical with the values for naturally occurring **1**.

**Figure 5:**
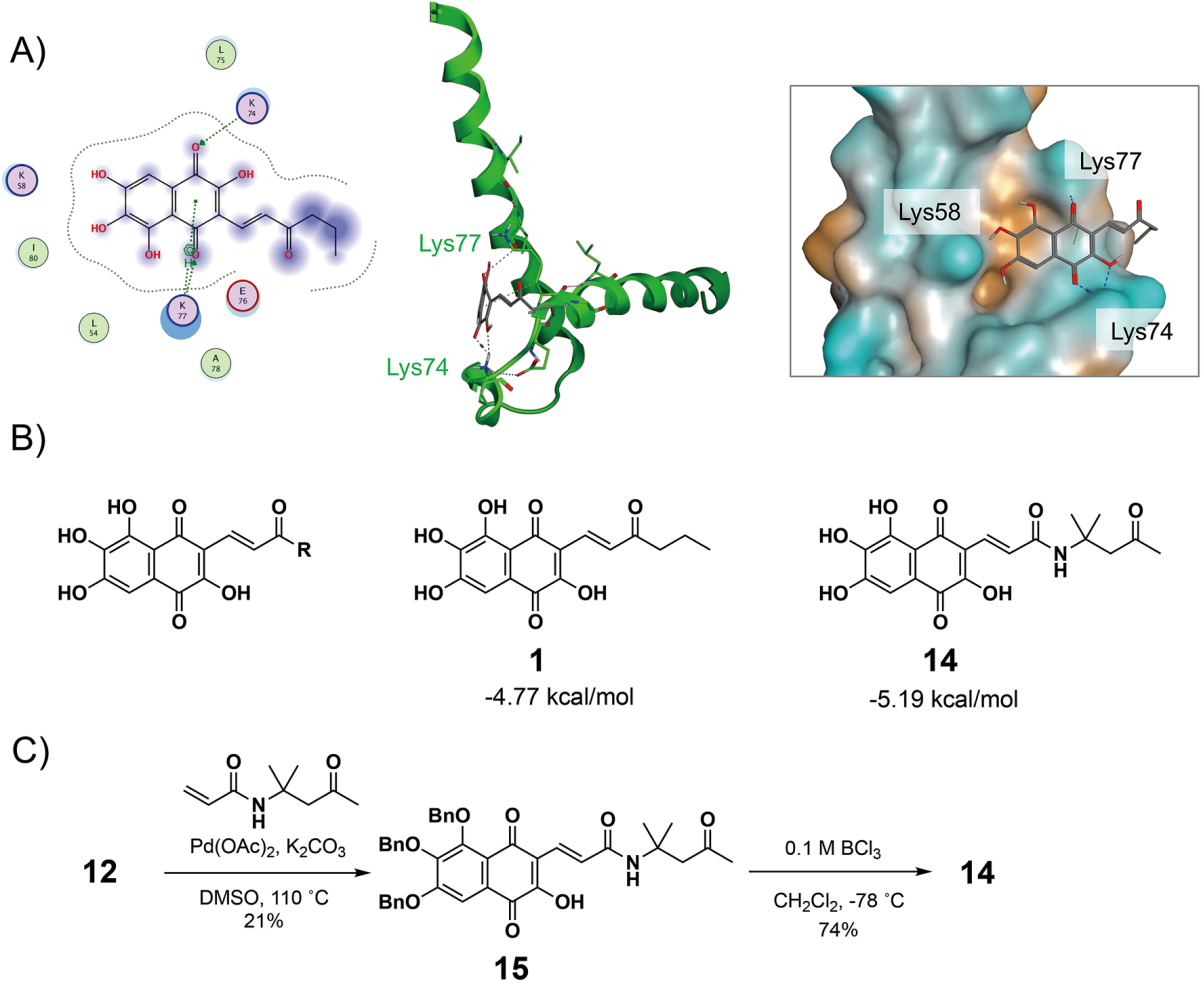
(**A**) Study of docking of **1** with Hes1. X-ray structure of the HLH domains of Hes1 dimers (PDB code: 2MH3) was used. (**B**) In silico screening. R substituents were examined from a linker database containing 3408 units and 26 commercially available compounds. (**C**) Synthesis of a derivative of **1**.

To obtain a derivative of **1** exhibiting stronger inhibitory activity, a docking simulation study was performed (Fig. 5A). The results of this study suggested that ketone units of lindbladione (**1**) interact with Lys74 and Lys77. Two phenolic hydroxy groups of **1** were found close to Lys58. Interestingly, CH/π interaction between **1** and Lys77 was also predicted. Next, we performed in silico screening of derivatives of **1**. R substituents of **1** were screened from a linker database containing 3408 units and 26 commercially available compounds (Fig. 5B). The calculations identified several compounds having lower energy than **1** (Supporting Information, Figure S1). We are interested in the effect of side chain amides in these compounds. A derivative **14** was synthesized in the same manner as B (Fig. 5C).

**Figure 6:**
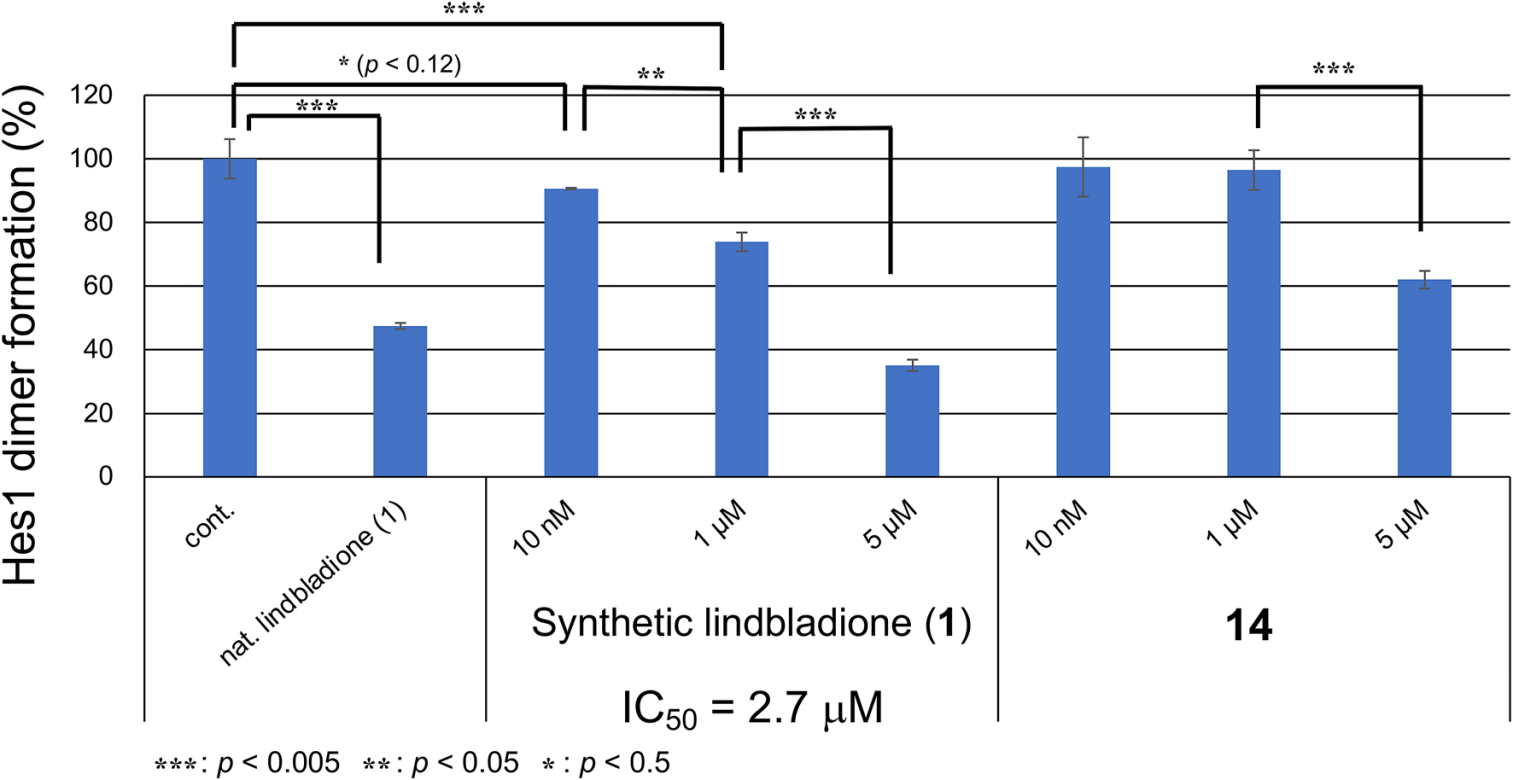
Inhibition of Hes1 dimer formation by isolated natural compound **1** and synthetic compounds **1** and **14**. n = 3; error bars indicate standard division; “cont.” indicates control (DMSO), “nat. lindbladione (**1**)” indicates natural product **1** (5 μM). *p* values were determined using the Student’s *t* test.

The inhibition of Hes1 dimer formation was investigated using our original Hes1 dimer plate assay (Fig. 6)^8^. We previously developed this plate assay. Hes1 was immobilized on the bottom of 96 well plate, then Cy3-labelled Hes1 was added. After making Hes1 dimer, compounds were added and incubated with Hes1. If compounds were inhibitors, fulorescence would be decreased (Fig. 2). Synthetic lindbladione (**1**) inhibited Hes1 dimer formation dose dependently, with an IC_50_ value of 2.7 μM, comparable to that of naturally occurring **1** (3.7 μM). The protected compounds **5**, **6**, and **8** did not exhibit inhibitory activity, which suggested that the phenolic hydroxy groups are important for this activity (Supporting Information, Figure S2). The amide derivative **14** exhibited inhibition, but the activity was not stronger than that of lindbladione (**1**). Indeed, there was lower energy conformation of **14** with Hes1 (− 5.1945 kcal/mol). However, the difference of molecular weight might result in lower energy. And also, the calculation results showed compound **14** binds to Hes1 with several forms (five main forms). The highest (unstable) energy was − 4.6542 kcal/mol, this is almost same as that of lindbladione (**1**) (− 4.7714 kcal/mol). This might be one of the reasons why Hes1 dimer inhibition of **14** was not as potent as **1**. In addition, the hydrophobic interaction of methyl on the side chain of **1** seems to be important. However, methyl ketone in **14** might be not efficient for suitable hydrophobic interaction.

**Figure 7:**
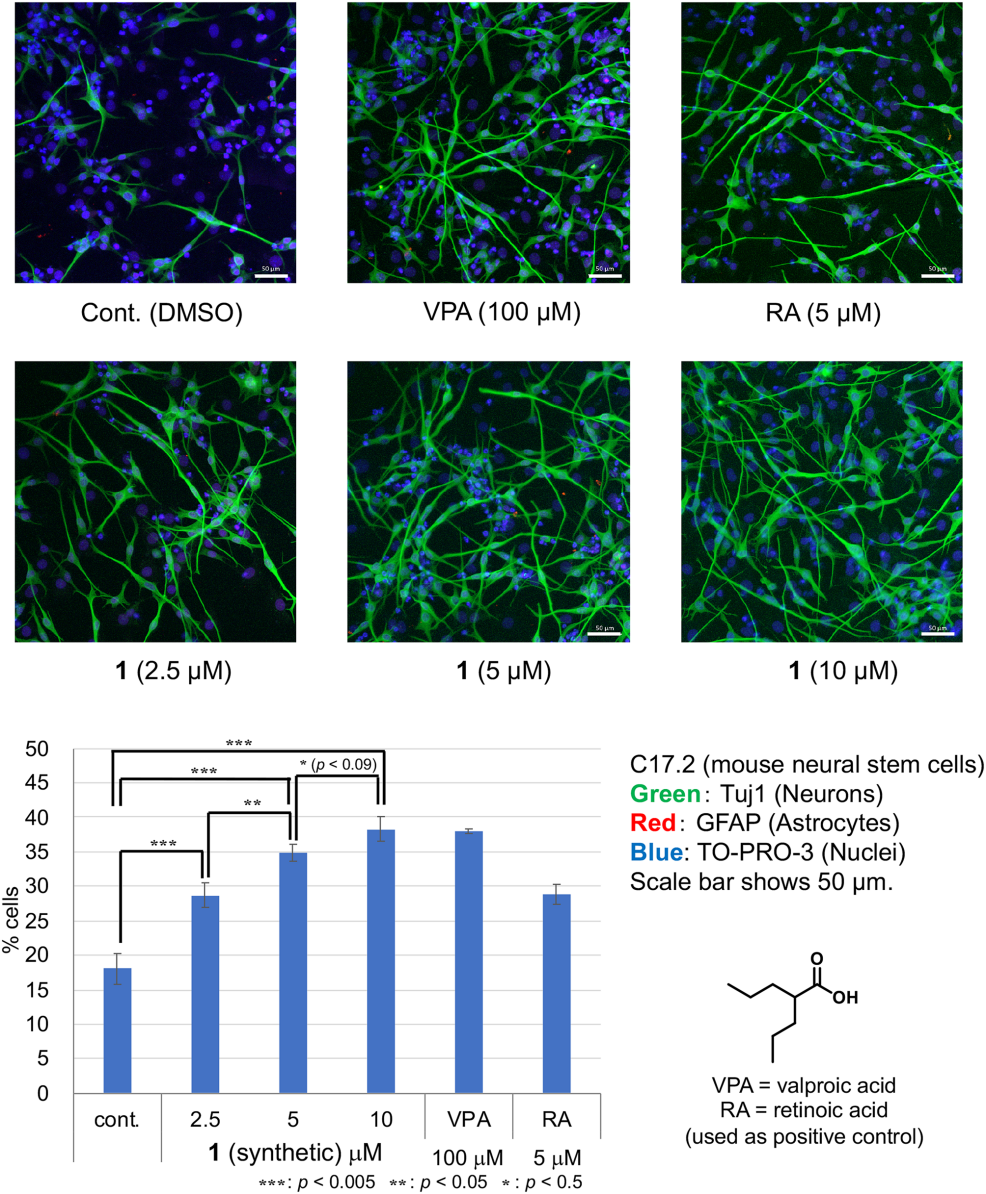
Effect of **1** (synthetic) on NSC differentiation into neurons after 4 days. Mouse C17.2 NSCs were used. Green: neurons; blue: nuclei; red: astrocytes. The number of neurons increased dose dependently following the addition of compound **1**. *p* values were determined using the Student’s *t* test.

Next, the effect of synthetic **1** on NSC differentiation was examined (Fig. 7). Mouse NSCs (C17.2) were treated with DMSO (control), valproic acid^20^ (100 μM, positive control), retinoic acid^21^ (5 μM, positive control), or synthetic **1**. After incubation for 4 days, the cells were immunostained (neurons: Tuj1, astrocytes: GFAP, nuclei: TO-PRO-3), and the number of neurons was determined. Synthetic **1** accelerated the differentiation of NSCs into neurons by 59% at 2.5 μM and 112% at 10 μM. As naturally occurring **1** induced a 25% increase in the number of neurons at 2.5 μM^14^, the results for synthetic **1** were comparable to those of naturally occurring **1**.

## Conclusion

In conclusion, total synthesis of the first Hes1 dimerization inhibitor, lindbladione (**1**), was accomplished. Synthetic **1** exhibited comparable Hes1 dimerization inhibition in our original Hes1 dimerization plate assay and accelerated NSC differentiation comparable to isolated **1**. As lindbladione (**1**) exhibited good selectivity against other basic helix-loop-helix transcriptional factors, such as the interaction between TCF and β-catenin (IC_50_ = 80.6 μM, Supporting Information, Figure S3), the final step in Wnt signaling, and there are few reports of Hes1 dimerization inhibitors, these results will be useful for evaluating other Hes1 inhibitors.

## Methods

### General experimental procedures

JEOL ECA600, ECZ400 and ECZ600 were used for recording spectra of nuclear magnetic resonance (NMR) spectra. JASCO FT/IR-4700 spectrometer was used for recording IR spectra. JEOL AccuTOF LC-plus JMS-T100LP mass spectrometer was used for obtaining HRMS spectra. JASCO an LC-2000 Plus series were used for high-performance liquid chromatography (HPLC).

### Plate assay for Hes1 dimer inhibitors

Hes1-bound microplate wells were constructed as reported before^8^ (Nunc Immobilizer Amino Plate, Thermo), and wells were incubated with 50 μL of Cy3-labeled-Hes1 in NET-N buffer (NET buffer (20 mm Tris–HCl, pH 7.5, 200 mm NaCl, 1 mm EDTA) containing 0.05% Nonidet P-40, ca. 7 mg/L) for 21 h at 4 °C. To remove extra the Cy3-proteins, the wells were washed twice with 200 μL of PBST. To the wells was added each compound solution (in NET-N buffer, 50 μL) and the plates were incubated for 1 h at room temperature (r.t.) in the dark. The wells were washed twice with 200 μL of PBST, then dried under reduced pressure for 1 h in the dark. The Cy3 dye was excited at 544 nm and emission was monitored at 590 nm using a microplate reader (Fluoroskan Ascent, Thermo Labsystems, Vantaa, Finland). The assays were carried out in three individual wells, and the mean value and SD were calculated.

### Neural stem cell differentiation assay

C17.2 cell line was purchased from European collection of cell cultures. C17.2 cells (2 × 10^5^ cells/mL) in proliferation medium were seeded on cover glass (Matsunami Glass) coated with fibronectin/laminin and poly-L-lysine. Cells were washed with D-MEM after incubation for 12 h, then medium was changed to differentiation medium to initiate differentiation of C17.2 cells with individual compounds for 4 days. Immunofluorescence staining method was reported before^11^. Proliferation medium: DMEM (DS Pharma Biomedical Co.) supplemented with 10% fetal bovine serum (BioWest) and 5% horse serum (Gibco), differentiation medium: Neurobasal Medium (Gibco) with 2% B-27 without vitamin A (Gibco) and 1% antibiotic–antimycotic (Gibco).

### In silico analysis and screening

Hes1 homodimer domain was constructed based on the structure of HLH domain taken from the Protein Data Bank (PDB) (PDB code: 2MH3) by using MOE (version 2016; Chemical Computing Group, Montreal, Canada). Water molecules in the crystal structure were removed. All hydrogen atoms were added and Amber all-atom charges were assigned for the whole protein. The initial 3D structures of lindbladione (**1**) were constructed using Build in MOE with standard geometric parameters. Then, fragment-based drug design was performed with lindbladione (**1**) as the parent nucleus to generate 3434 analogs using Fragment-based drug design in MOE. Lindbladione (**1**) and 3434 analogs were minimized using Energy Minimize in MOE with the Amber force field until the root-mean-square (rms) energy gradient was less than 0.001 kcal mol^−1^ Å^−1^. The molecular docking simulations, lindbladione (**1**) and 3434 analogs for Hes1 homodimer domain, were performed using the rigid methods in MOE-Dock.

### Statistical analysis

All measurements were performed in triplicate and were reported as the mean ± standard deviation (SD). A *p* value was determined by the Student’s *t* test. Variables with a *p* value of less than 0.05 were considered statistically significant.

## Supplementary information

Supplementary Information
