# Supplementary material for: Total synthesis of lindbladione, a Hes1 dimerization inhibitor and neural stem cell activator isolated from Lindbladia tubulina
Source: Sci Rep. 2020 Dec 8;10:21433. doi: 10.1038/s41598-020-78524-7 (PMC7722756; doi:10.1038/s41598-020-78524-7)
Supplement: Supplementary file 1 — Supplementary Information [file 41598_2020_78524_MOESM1_ESM.pdf]

Supporting Information

Total Synthesis of Lindbladione, a Hes1 Dimerization Inhibitor and Neural Stem Cell Activator Isolated from *Lindbladia tubulina*

Midori A. Arai,[1*] *Yuna Makita,*[2] *Yumi Yamaguchi,*[2] *Haruka Kawano,*[2] *Akiko Suganami,*[3] *Yutaka Tamura,*[3] *Masami Ishibashi*[2*]

[1] Department of Biosciences and Informatics, Faculty of Science and Technology, Keio University, 3-14-1 Hiyoshi, Kohoku-ku, Yokohama 223-8522, Japan

[2] Graduate School of Pharmaceutical Sciences, Chiba University, 1-8-1 Inohana, Chuo-ku, Chiba 260-8675, Japan

[3] Graduate School of Medicine, Chiba University, 1-8-1 Inohana, Chuo-ku, Chiba 260-8670, Japan

Methyl 2-(3,4,5-trimethoxyphenyl)acetate (3). To a solution of 3,4,5-trimethoxyphenylacetic acid (5310 mg, 22.1 mmol) in MeOH (22.1 mL) was added thionyl chloride (1.7 mL, 24.3 mmol) dropwise. The reaction mixture was stirred for 6 h at 50 °C. After concentration, the mixture was diluted with CH_2Cl_2. The organic layer was washed with saturated aq. $NaHCO_3$ and brine, dried over Na_2SO_4. After filtration and concentration, the resulting residue was purified by silica gel column chromatography (hexane/AcOEt = 2/1) to afford **3** (5393 mg, quant.). ^{1}H-NMR (400 MHz, $CDCl_3$) δ 6.48 (s, 2H), 3.84 (s, 6H), 3.81 (s, 3H), 3.69 (s, 3H), 3.54 (s, 2H); ^{13}C-NMR (101 MHz, $CDCl_3$) δ 171.9, 153.1, 137.0, 129.3, 106.2, 60.7, 56.0, 52.0, 41.3; IR (ATR) 2943, 2839, 1733, 1592, 1508, 1460, 1423, 1321, 1241, 1124, 1006, 830, 787, 652, 633, 613 cm^{-1}; ESI-MS *m/z* 241 [M+H]$^{+}$.

Methyl 2-(2-acetyl-3,4,5-trimethoxyphenyl)acetate (4). To a solution of **3** (393 mg, 1.6 mmol) in acetic anhydride (8.2 mL) was added copper (II) trifluoromethane sulfonate (118 mg, 0.33 mmol). The reaction mixture was stirred for 1 h at 50 °C and diluted with CH_2Cl_2. The mixture was washed with H_2O and saturated aq. $NaHCO_3$ three times. The organic layer was dried over Na_2SO_4. After filtration and concentration, the resulting residue was purified by silica gel column chromatography (hexane/AcOEt = 3/1) to afford **4** (416 mg, 1.5 mmol, 90%). ^{1}H-NMR (400 MHz, $CDCl_3$) δ 6.51 (s, 1H), 3.90 (s, 3H), 3.85 (s, 3H), 3.85 (s, 3H), 3.67 (s, 3H), 3.65 (s, 2H), 2.51 (s, 3H); ^{13}C-NMR (151 MHz, $CDCl_3$) δ 203.7, 171.8, 154.6, 152.1, 140.9, 128.5, 127.7, 110.2, 61.5, 60.8, 56.0, 52.0, 38.4, 32.2; IR (ATR) 1593, 1319, 1152, 666, 644, 614 cm^{-1}; ESI-HRMS *m/z* calcd for $C_{14}H_{18}NaO_6$ 305.1001, found 305.1005 [M+Na]$^{+}$.

Methyl 2-(2-acetyl-3,4,5-tris(benzyloxy)phenyl)acetate (9) and **methyl 2-(2-acetyl-3,4-bis(benzyloxy)-5-methoxyphenyl)acetate (10).** To a solution of **4** (1120 mg, 3.97 mmol) in CH_2Cl_2 (19.8 mL) was added 1 M BBr_3 in CH_2Cl_2 (11.9 mL, 11.9 mmol) at -78 °C. The reaction mixture was stirred for 30 min, then allowed to warm to 0 °C and stirred for 30 min. After stirring additional 1 h at room temperature, the mixture was quenched with MeOH and concentrated then the resulting residue was purified by silica gel column chromatography (hexane/AcOEt = 1/1) to afford a mixture of trihydroxy- and dihydroxyphenyl compounds in the ratio of 4:1. To a solution of this mixture (902 mg) and K_2CO_3 (3290 mg, 23.8 mmol) in DMF (31.7 mL) was added benzyl bromide (4.24

mL, 35.7 mmol) dropwise. The reaction mixture was stirred for 13 h at 40 °C, then quenched with H_2O and extracted with AcOEt. The organic layer was washed with H_2O and brine, dried over Na_2SO_4. After filtration and concentration, the resulting residue was purified by silica gel column chromatography (hexane/AcOEt = 7/1) to afford **9** (923 mg, 1.81 mmol, 46% in 2 steps) and **10** (204 mg, 0.470 mmol, 12% in 2 steps). Spectral data of **9**: ^{1}H-NMR (400 MHz, $CDCl_3$) δ 7.26-7.43 (m, 15H), 6.65 (s, 1H), 5.11 (s, 2H), 5.04 (s, 2H), 5.03 (s, 2H), 3.67 (s, 3H), 3.65 (s, 2H), 2.48 (s, 3H); ^{13}C-NMR (151 MHz, $CDCl_3$) δ 203.96, 171.74, 153.89, 152.77, 151.17, 140.58, 137.09, 137.02, 136.78, 136.21, 129.53, 128.62, 128.59, 128.51, 128.45, 128.31, 128.16, 128.10, 127.80, 127.73, 127.69, 127.56, 127.41, 112.04, 108.85, 76.44, 75.60, 71.12, 70.94, 52.03, 38.36, 32.53; IR (ATR) 3032, 1739, 16889, 1593, 1497, 1454, 1424, 1318, 1274, 1156, 1098, 1003, 740, 698, 631, 613 cm^{-1}; ESI-HRMS *m/z* calcd for $C_{32}H_{30}NaO_6$ 533.1940, found 533.1993 $[M+Na]^+$.

5,6,7-Tris(benzyloxy)-2-hydroxynaphthalene-1,4-dione (11). To a solution of **9** (923 mg, 1.81 mmol) in MeOH (60.3 mL) was added sodium methoxide (2.89 mL, 5 M in MeOH). The reaction mixture was refluxed for 3 h. Following this period, the flask was opened to the atmosphere and the mixture was stirred for 18 h at room temperature. Then the reaction was quenched with 1 *N* HCl. The mixture was extracted with CH_2Cl_2. The organic layer was dried over Na_2SO_4. After filtration and concentration, the resulting residue was purified by silicagel column chromatography (hexane/AcOEt = 1/1) to afford **11** (579 mg, 1.18 mmol, 65%). ^{1}H-NMR (400 MHz, $CDCl_3$) δ 7.27-7.58 (m, 16H), 6.20 (s, 1H), 5.21 (s, 2H), 5.11 (s, 2H), 5.03 (s, 2H); ^{13}C-NMR (101 MHz, $CDCl_3$) δ 184.01, 181.49, 156.02, 154.59, 153.54, 149.37, 136.59, 135.34, 129.15, 128.71, 128.50, 128.39, 128.33, 127.64, 126.55, 120.44, 112.19, 107.86, 76.03, 75.86, 71.15; IR (ATR) 2309, 1508, 1317, 1032, 675, 642, 614 cm^{-1}; ESI-HRMS *m/z* calcd for $C_{31}H_{24}NaO_6$ 515.1471, found 515.1474. $[M+Na]^+$.

5,6,7-Tris(benzyloxy)-2-hydroxy-3-iodonaphthalene-1,4-dione (12). To a solution of **11** (150 mg, 0.304 mmol) in CH_2Cl_2 (12.2 mL) was added *N*-iodosuccinimide (75.3 mg, 0.335 mmol). The reaction mixture was refluxed for 3 h, then added H_2O and extracted with CH_2Cl_2. The organic layer was washed with H_2O, dried over Na_2SO_4. After filtration and concentration, the resulting residue was purified by silica gel column chromatography (CH_2Cl_2) to afford **12** (139 mg, 0.224 mmol, 74%). ^{1}H-NMR (400 MHz,

$CDCl_3$) δ 7.27-7.58 (m, 16H), 5.21 (s, 2H), 5.09 (s, 2H), 5.05 (s, 2H); ^{13}C-NMR (151 MHz, $CDCl_3$) δ 177.14, 176.93, 158.21, 156.33, 154.34, 149.13, 136.52, 136.37, 135.19, 129.12, 128.77, 128.59, 128.39, 128.36, 128.34, 128.27, 127.70, 125.82, 119.45, 108.32, 94.83, 76.03, 75.83, 71.26; IR (ATR) 3648, 2981, 2310, 1508, 1318, 1033, 685, 614 cm^{-1}; ESI-HRMS *m/z* calcd for $C_{31}H_{23}O_6$ 618.0539, found 618.0571. $[M-H]^-$.

(*E*)-5,6,7-tris(benzyloxy)-2-hydroxy-3-(3-oxohex-1-en-1-yl)naphthalene-1,4-dione (13) To a solution of **12** (139 mg, 0.224 mmol), K_2CO_3 (155 mg, 1.12 mmol) and palladium (II) acetate (10.1 mg, 44.8 μmol) in H_2O/DMSO (1:1, 2.24 mL) was added 1-hexene-3-one (0.145 mL, 1.12 mmol, >90% stabilized with 0.5% 4-methoxyphenol). The reaction mixture was stirred at 110 °C for 1.5 h, then acidified with 25% H_3PO_4 and extracted with CH_2Cl_2. The organic layer was washed with H_2O and dried over Na_2SO_4. After filtration and concentration, the resulting residue was purified by silica gel column chromatography (CH_2Cl_2) to afford **13** (124 mg, 0.210 mmol, 94%). ^{1}H-NMR (600 MHz, $CDCl_3$) δ 7.78 (d, J = 17.2 Hz, 1H), 7.23-7.58 (m, 18H), 5.21 (s, 3H), 5.12 (s, 3H), 5.06 (s, 3H), 2.68 (t, J = 7.2 Hz, 2H), 1.67-1.72 (m, 2H), 0.97 (t, J = 7.2 Hz, 3H); ^{13}C-NMR (151 MHz, $CDCl_3$) δ 201.91, 181.81, 180.40, 156.29, 153.94, 153.09, 149.69, 136.54, 136.41, 135.21, 134.46, 130.93, 129.10, 128.75, 128.73, 128.60, 128.55, 128.43, 128.34, 127.67, 126.37, 120.40, 116.92, 107.55, 75.97, 75.86, 71.20, 42.33, 17.77, 13.84; IR (ATR) 2963, 1648, 1572, 1317, 1033, 698, 647, 631, 614 cm^{-1}; ESI-HRMS *m/z* calcd for $C_{37}H_{31}O_7$ 587.2070, found 587.2013 $[M-H]^-$.

Lindbladione (1). To a solution of **13** (11.2 mg, 19.0 μmol) in CH_2Cl_2 (951 μL) was added 0.1 M BCl_3 in CH_2Cl_2 (856 μL, 85.6 μmol) at -78 °C. The reaction mixture was stirred for 10 min, then added $NaHCO_3$ (144 mg). The mixture was diluted with MeOH and filtered through Celite. After concentration, the resulting residue was purified by ODS column chromatography (MeOH 40%) to afford **1**. IR (ATR) 3410, 1649, 1437, 1313, 1019, 9523 703, 620, 609 cm^{-1}; ESI-HRMS *m/z* calcd for $C_{16}H_{13}O_7$ 317.0617, found 317.0661 $[M-H]^-$.

(*E*)-N-(2-methyl-4-oxopentan-2-yl)-3-(6,7,8-tris(benzyloxy)-3-hydroxy-1,4-dioxo-1,4-dihydronaphthalen-2-yl)acrylamide (15) To a solution of **12** (31.3 mg, 50.6 μmol), K_2CO_3 (34.9 mg, 253 μmol) and palladium (II) acetate (2.3 mg, 10.1 μmol) in DMSO

(506 μL) was added diacetone acrylamide (42.8 mg, 253 μmol, stabilized with 4-methoxyphenol). The reaction mixture was stirred at 110 °C for 1.5 h, then acidified with 25% H_3PO_4 and extracted with CH_2Cl_2. The organic layer was washed with H_2O and dried over Na_2SO_4. After filtration and concentration, the resulting residue was purified by silica gel column chromatography (CH_2Cl_2) to afford **15** (7.1 mg, 10.8 μmol, 21%). ^{1}H-NMR (400 MHz, CD_3OD) δ 7.77 (d, *J* = 15.6 Hz, 1H), 7.24-7.58 (m, 15H), 7.06 (d, *J* = 15.6 Hz, 1H), 6.00 (s, 1H), 5.20 (s, 2H), 5.10 (s, 2H), 5.03 (s, 2H), 3.04 (s, 2H), 2.13 (s, 3H), 1.47 (s, 6H); IR (ATR) 2927, 1321, 618, 604 cm^{-1}; ESI-HRMS *m/z* calcd for $C_{40}H_{36}NO_8$ 658.2441, found 658.2487 $[M-H]^-$.

(*E*)-N-(2-methyl-4-oxopentan-2-yl)-3-(3,6,7,8-tetrahydroxy-1,4-dioxo-1,4-dihydronaphthalen-2-yl)acrylamide (14)

To a solution of **15** (8.9 mg, 13.5 μmol) in CH_2Cl_2 (675 μL) was added 0.1 M BCl_3 in CH_2Cl_2 (546 μL, 607 μmol) at -78 °C. The reaction mixture was stirred for 10 min, then added $NaHCO_3$ (102 mg). The mixture was diluted with MeOH and filtered through Celite. After concentration, the resulting residue was purified by ODS column chromatography (MeOH 40%) to afford **14** (3.9 mg, 10.0 μmol, 74%). ^{1}H-NMR (400 MHz, CD_3OD) δ 7.92 (d, *J* = 15.6 Hz, 1H), 7.21 (d, *J* = 15.6 Hz, 1H), 7.01 (s, 1H), 3.07 (s, 2H), 2.11 (s, 3H), 1.40 (s, 6H); ^{13}C-NMR (151 MHz, CD_3OD) δ 210.71, 189.47, 184.93, 175.19, 171.92, 170.18, 151.49, 149.14, 141.30, 134.89, 124.90, 118.16, 113.58, 112.15, 108.45, 64.42, 52.40, 31.63, 28.01; IR (ATR) 1366, 633, 618 cm^{-1} ; ESI-HRMS *m/z* calcd for $C_{19}H_{18}NO_8$ 388.1032, found 388.1064 $[M-H]^-$.

Table 1 NMR data of synthetic and natural **1**.

position	^{1}H-NMR in CD_3OD		^{13}C-NMR in CD_3OD	
	synthetic **1** (400 MHz)	natural **1** (600 MHz)	synthetic **1** (150 MHz)	natural **1** (150 MHz)
1			184.13	184.40
2			176.39	176.49
3			113.34	113.26
4			189.63	189.63
5			151.71	151.67
6			140.59	140.59
7			149.43	149.55
8	7.04 (s)	7.05 (s)	108.63	108.78
9			125.09	124.99
10			112.26	112.27
11	8.14 (d, 16.0)	8.13 (d, 16.0)	140.59	140.59
12	7.52 (d, 16.0)	7.52 (d, 16.0)	122.54	122.50
13			206.39	206.40
14	2.60 (t, 7.5)	2.60 (t, 7.5)	42.44	42.41
15	1.62-1.71 (m)	1.62-1.71 (m)	20.18	20.17
16	0.97 (t, 7.5)	0.97 (t, 7.5)	14.31	14.30

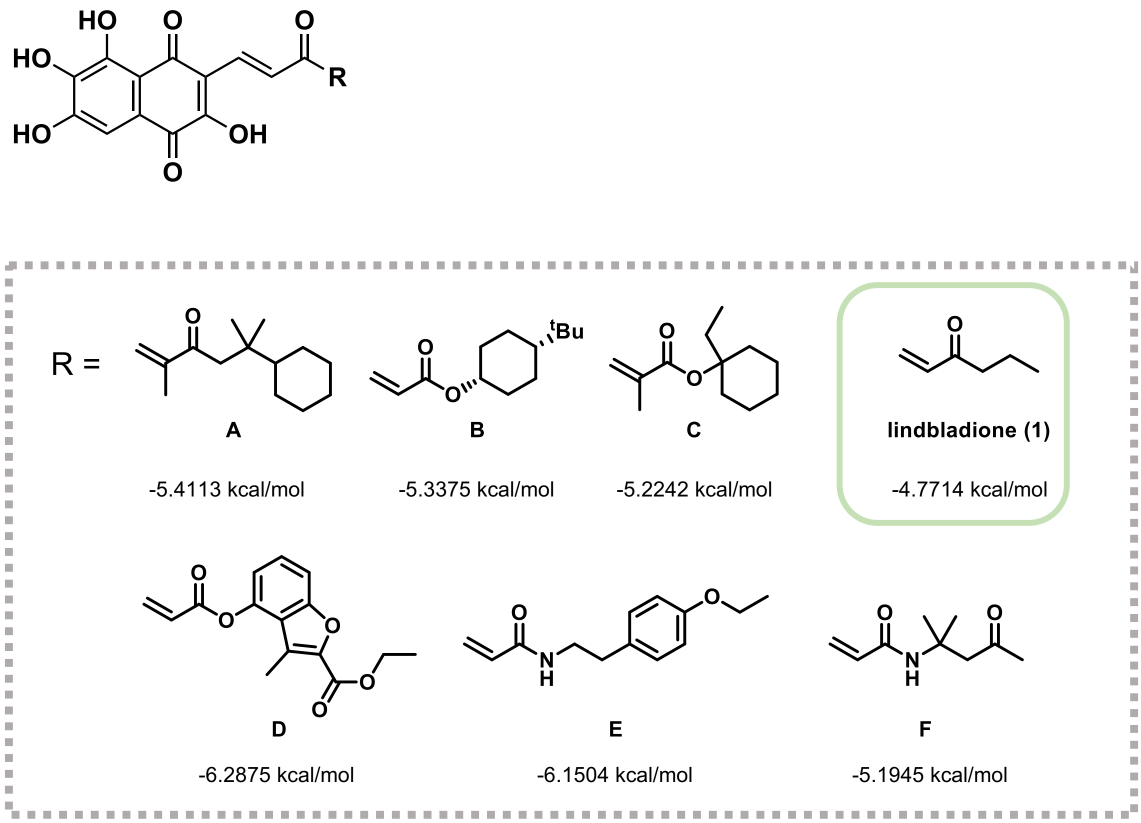

Figure S1 Docking energy of lindbladione derivatives.

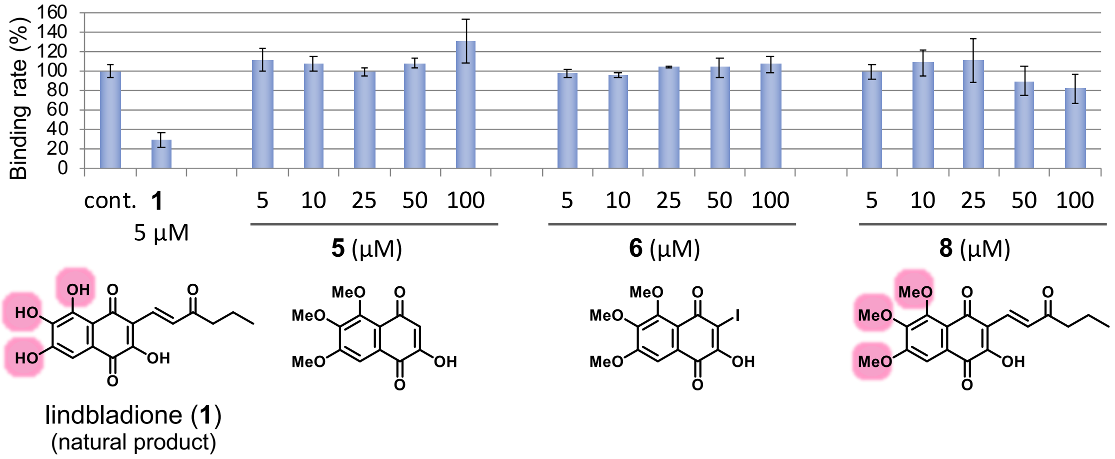

Figure S2 Protected lindbladione derivatives didn't show Hes1 dimer inhibition.

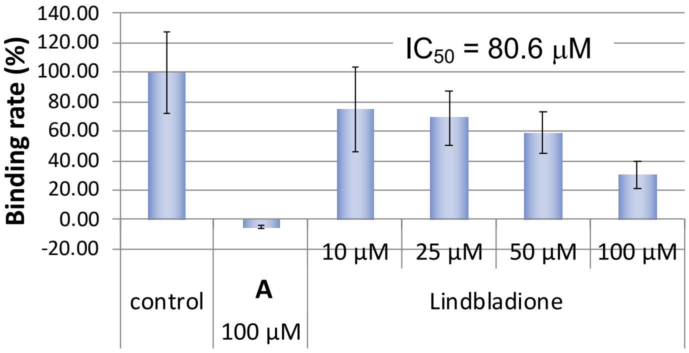

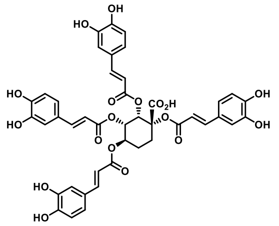

A: 1,3,4,5-tetra-*O*-caffeoylquinic acid

Figure S3 Lindbladione (**1**) show moderate inhibition of TCF/β-catenin complex.[ref]

Ref. Arai, M. A., Ishikawa, N., Tanaka, M., Uemura, K., Sugimitsu, N., Koyano, T., Kowithayakorn, T., Ishibashi, M. *Chem. Sci.* 7, 1514-1520 (2016).

3

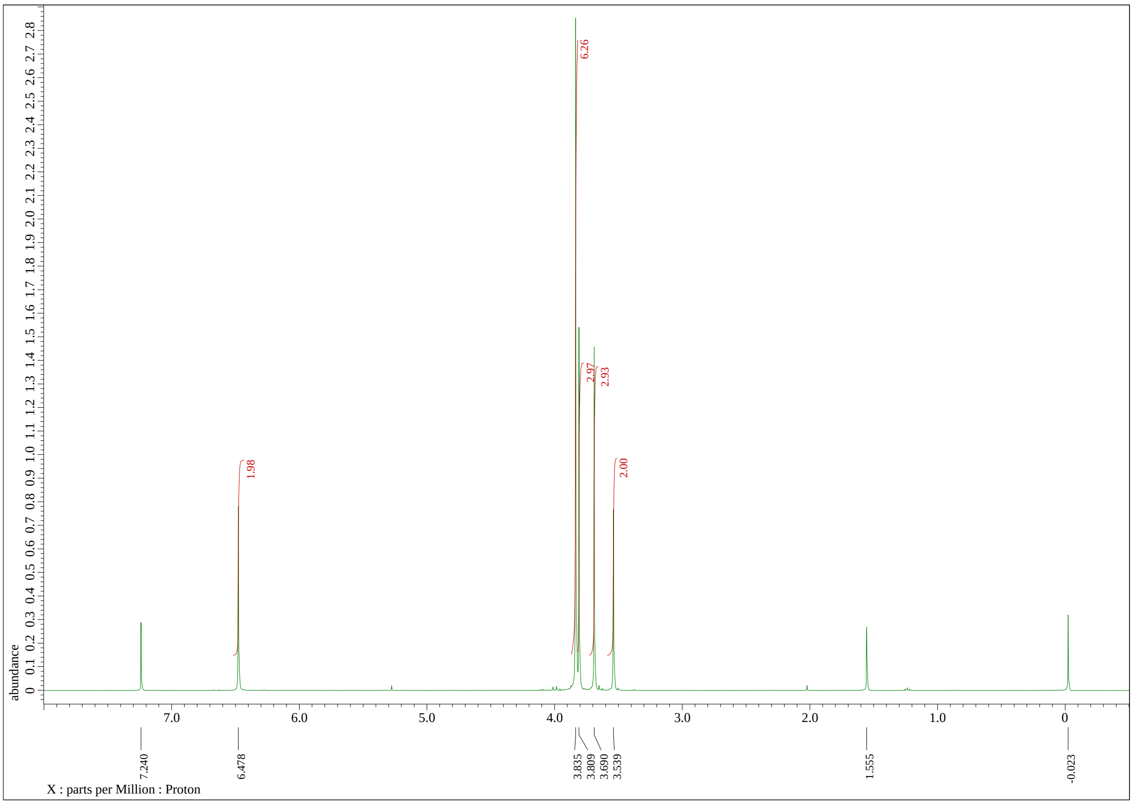

abundance
7.0
6.0
5.0
4.0
3.0
2.0
1.0
0
1.98
6.26
2.97
2.93
2.00
7.240
6.478
3.835
3.809
3.690
3.539
1.555
-0.023
X : parts per Million : Proton

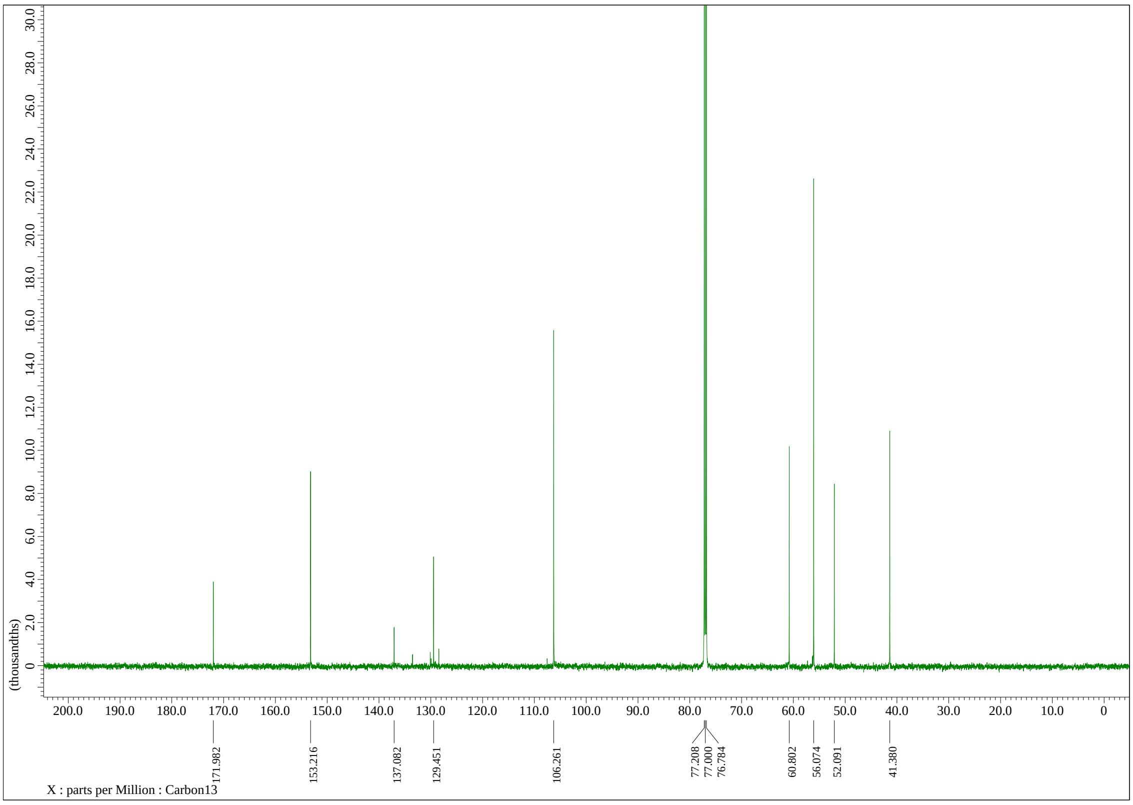

(thousandths)
171.982
153.216
137.082
129.451
106.261
77.208
77.000
76.784
60.802
56.074
52.091
41.380
X : parts per Million : Carbon13

4

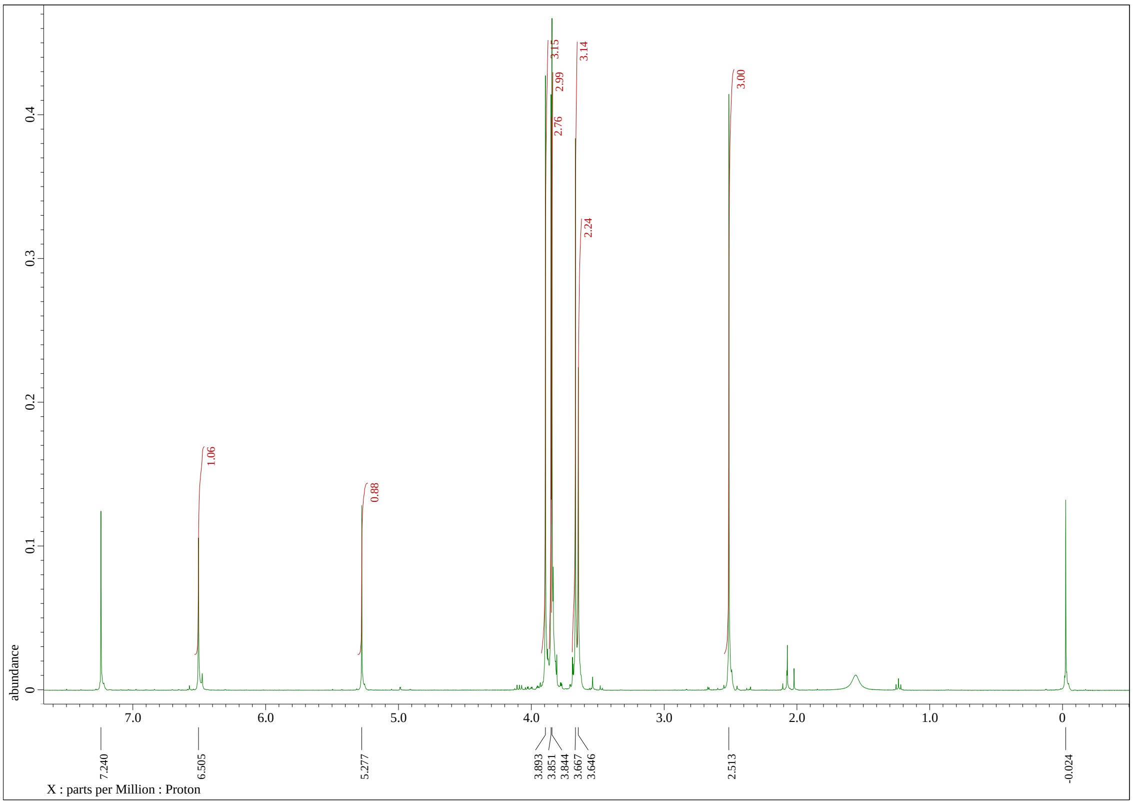

abundance
X : parts per Million : Proton

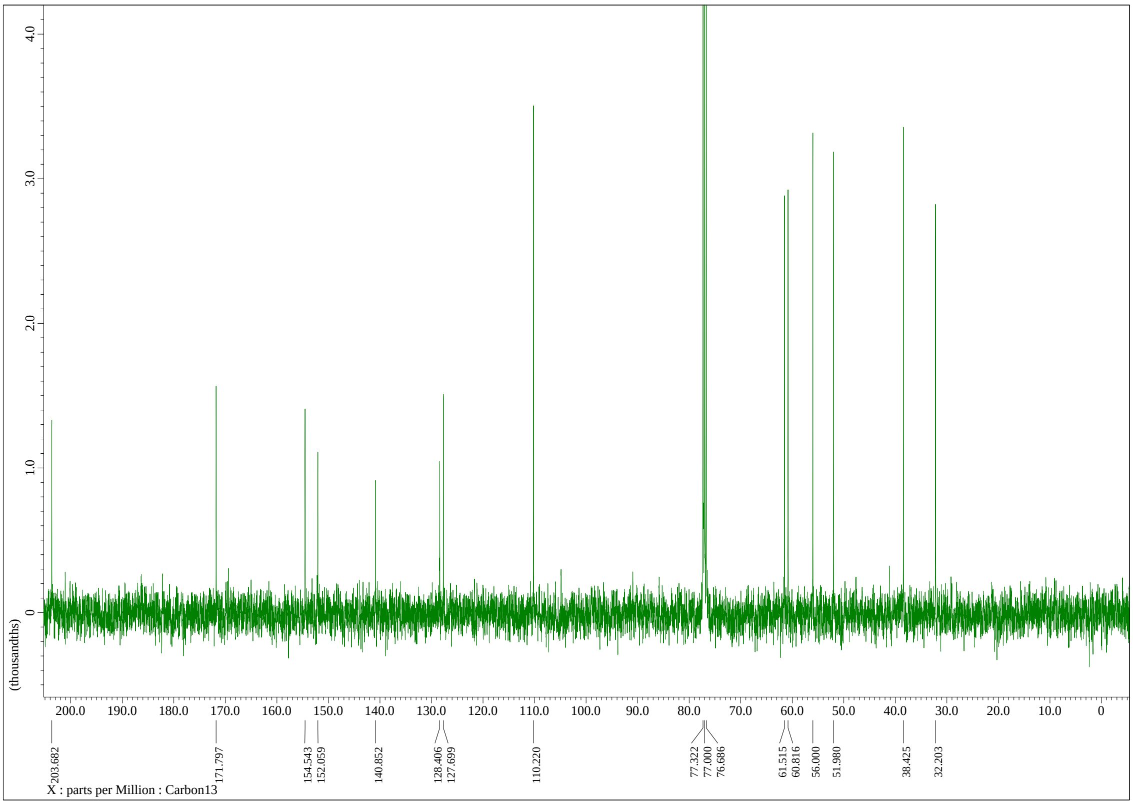

(thousandths)
X : parts per Million : Carbon13

9

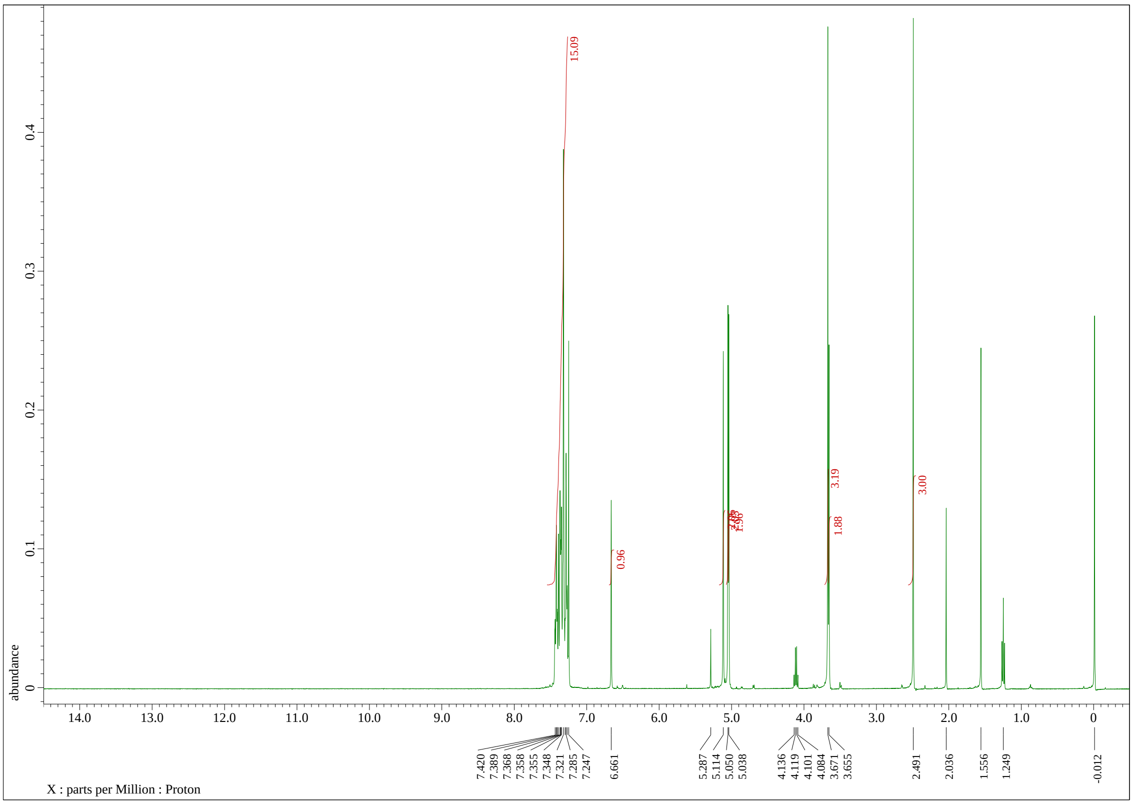
abundance
X : parts per Million : Proton
15.09
0.96
3.19
1.88
3.00
7.420
7.389
7.368
7.358
7.355
7.348
7.321
7.285
7.247
6.661
5.287
5.114
5.050
5.038
4.136
4.119
4.101
4.084
3.671
3.655
2.491
2.036
1.556
1.249
-0.012

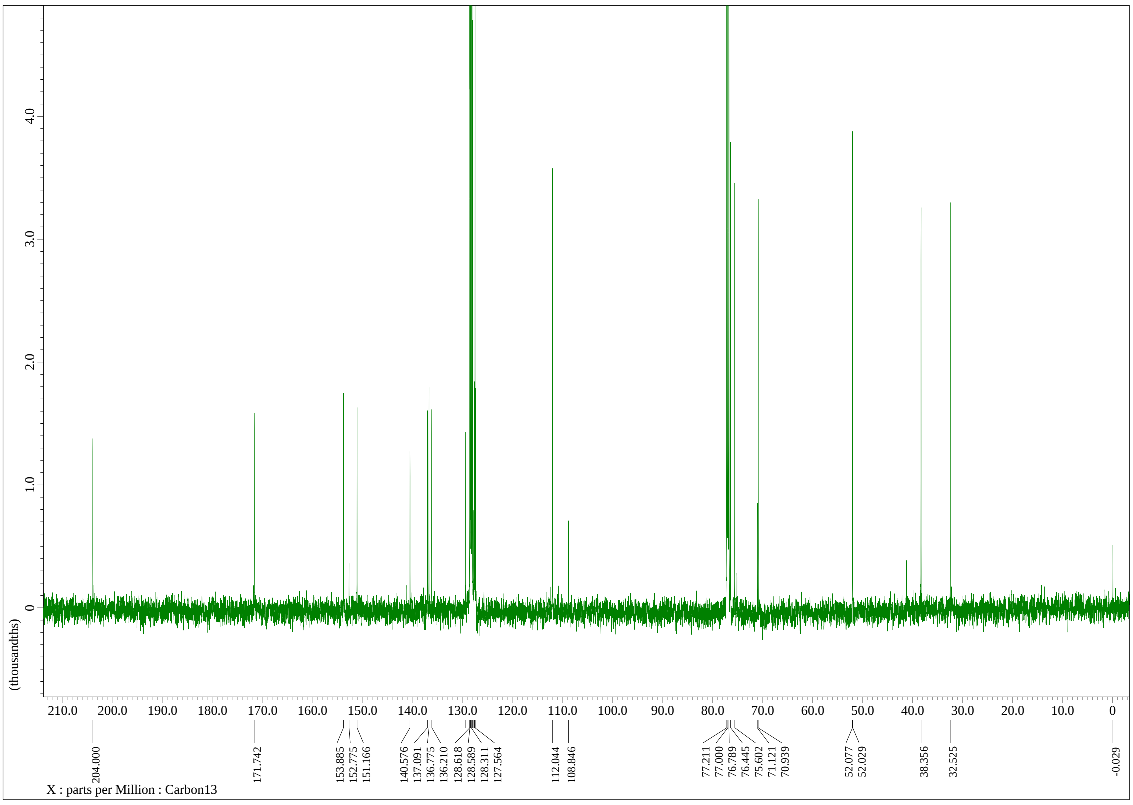
(thousandths)
X : parts per Million : Carbon13
204.000
171.742
153.885
152.775
151.166
140.576
137.091
136.775
136.210
128.618
128.589
128.311
127.564
112.044
108.846
77.211
77.000
76.789
76.445
75.602
71.121
70.939
52.077
52.029
38.356
32.525
-0.029

10

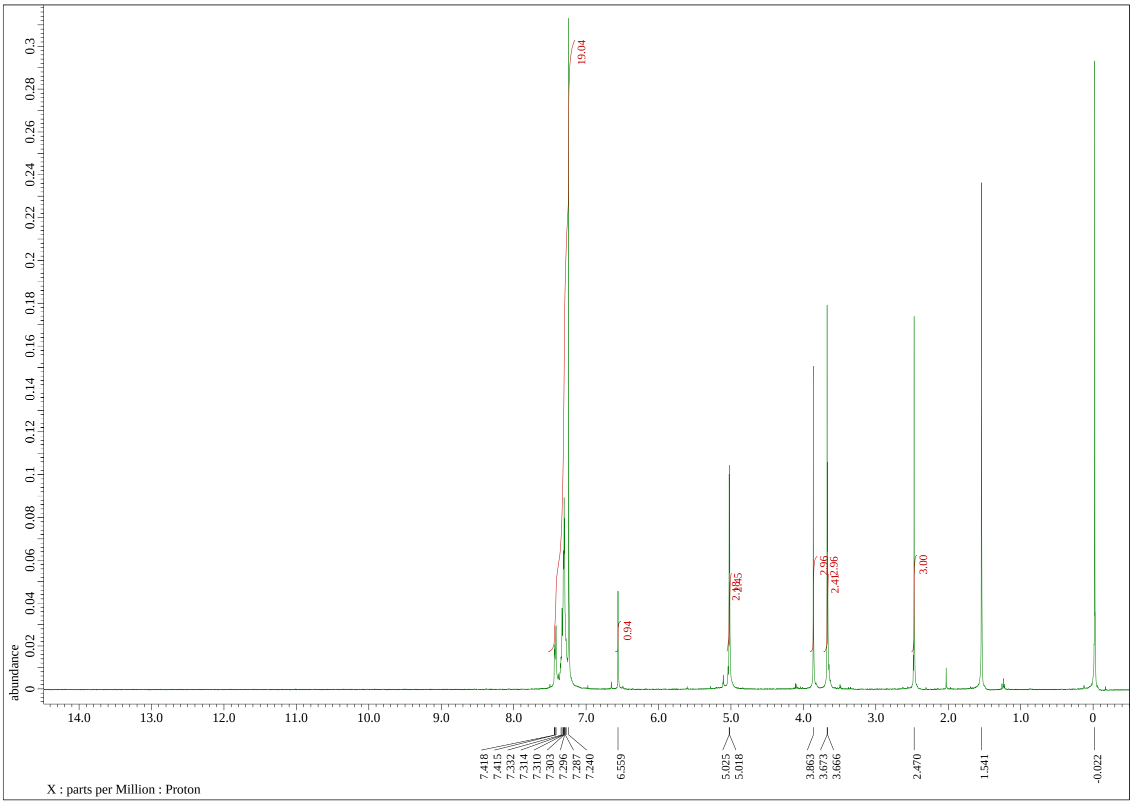

abundance
X : parts per Million : Proton
7.418 7.415 7.332 7.314 7.310 7.303 7.296 7.287 7.240
6.559
5.025 5.018
3.863 3.673 3.666
2.470
1.541
-0.022
19.04
0.94
3.00

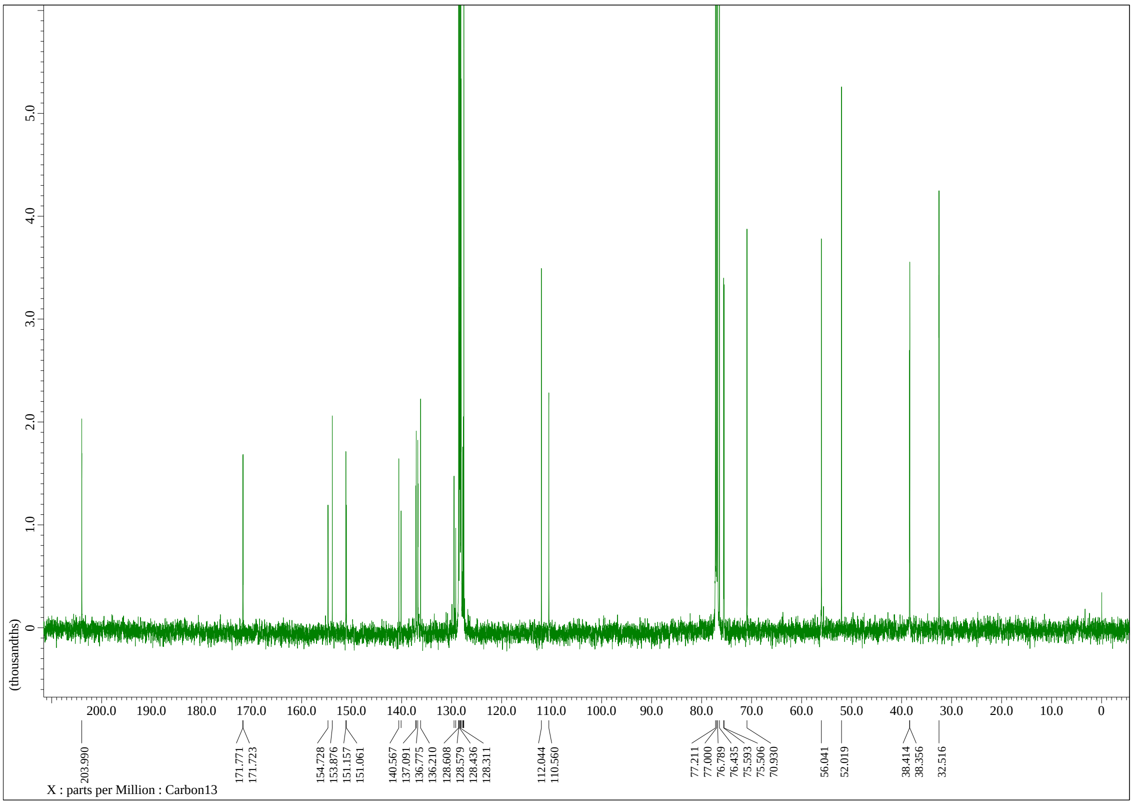

(thousandths)
X : parts per Million : Carbon13
203.990
171.771 171.723
154.728 153.876 151.157 151.061
140.567 137.091 136.775 136.210 128.608 128.579 128.436 128.311
112.044 110.560
77.211 77.000 76.789 76.435 75.593 75.506 70.930
56.041 52.019
38.414 38.356 32.516

11

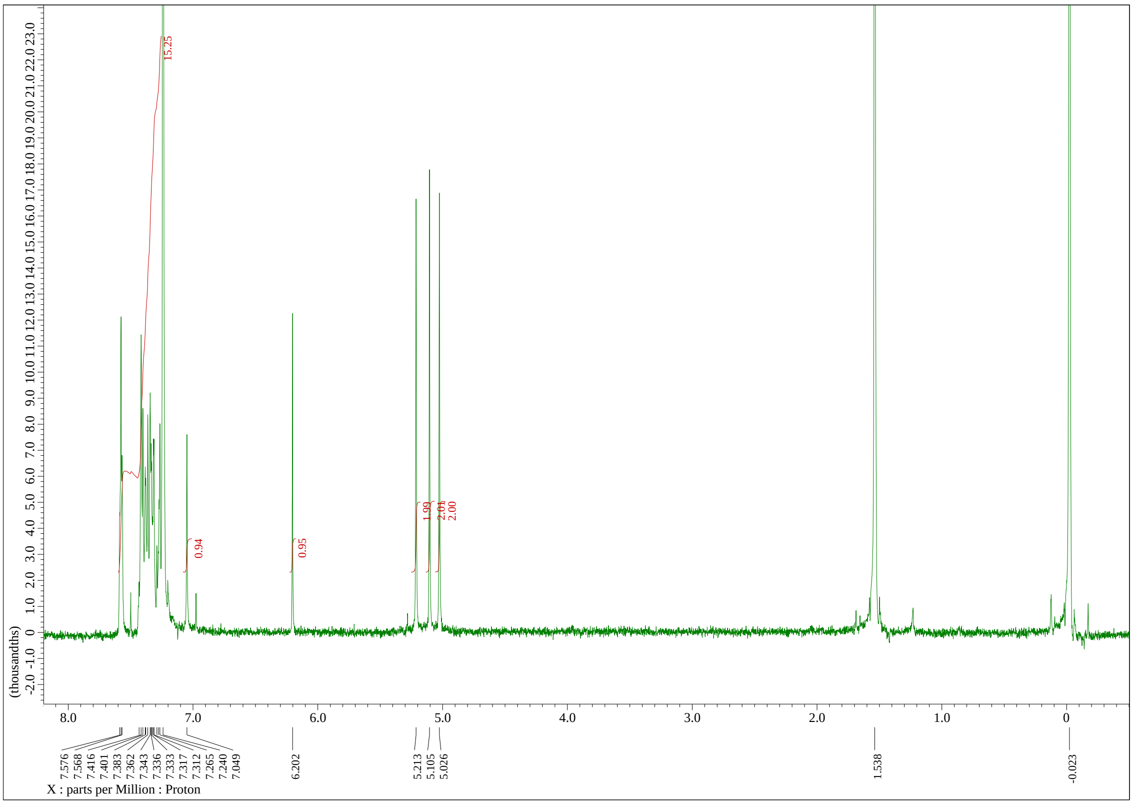
(thousandths)
X : parts per Million : Proton
15.25
0.94
0.95
1.99
2.01
2.00
7.576
7.568
7.416
7.401
7.383
7.362
7.343
7.336
7.333
7.317
7.312
7.265
7.240
7.049
6.202
5.213
5.105
5.026
1.538
-0.023

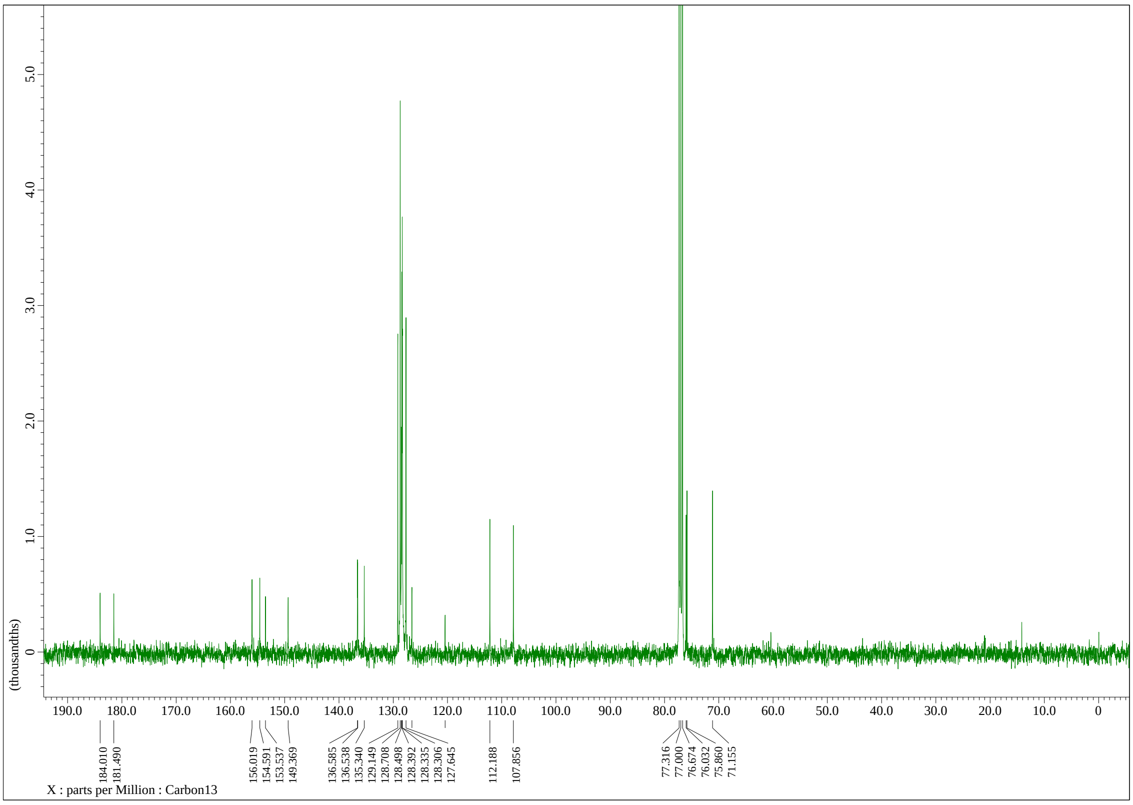
(thousandths)
X : parts per Million : Carbon13
184.010
181.490
156.019
154.591
153.537
149.369
136.585
136.538
135.340
129.149
128.708
128.498
128.392
128.335
128.306
127.645
112.188
107.856
77.316
77.000
76.674
76.032
75.860
71.155

12

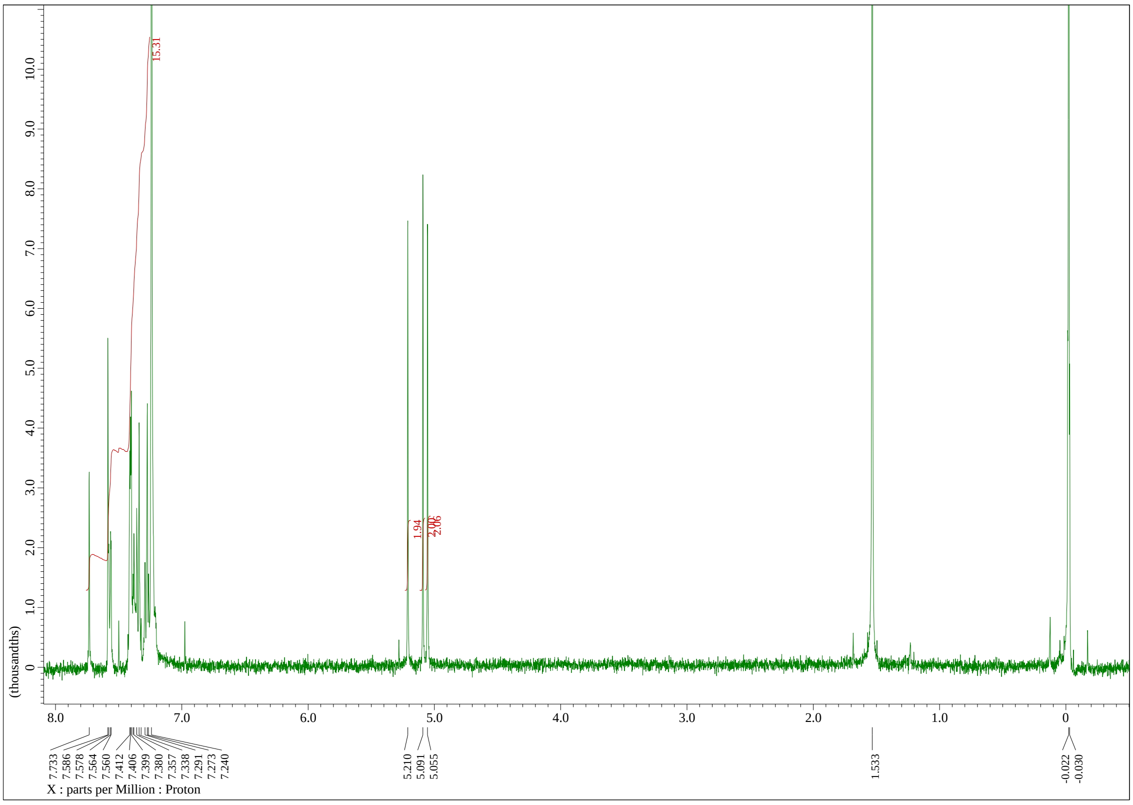

15.31
1.94
2.06
(thousandths)
7.733
7.586
7.578
7.564
7.560
7.412
7.406
7.399
7.380
7.357
7.338
7.291
7.273
7.240
5.210
5.091
5.055
1.533
-0.022
-0.030
X : parts per Million : Proton

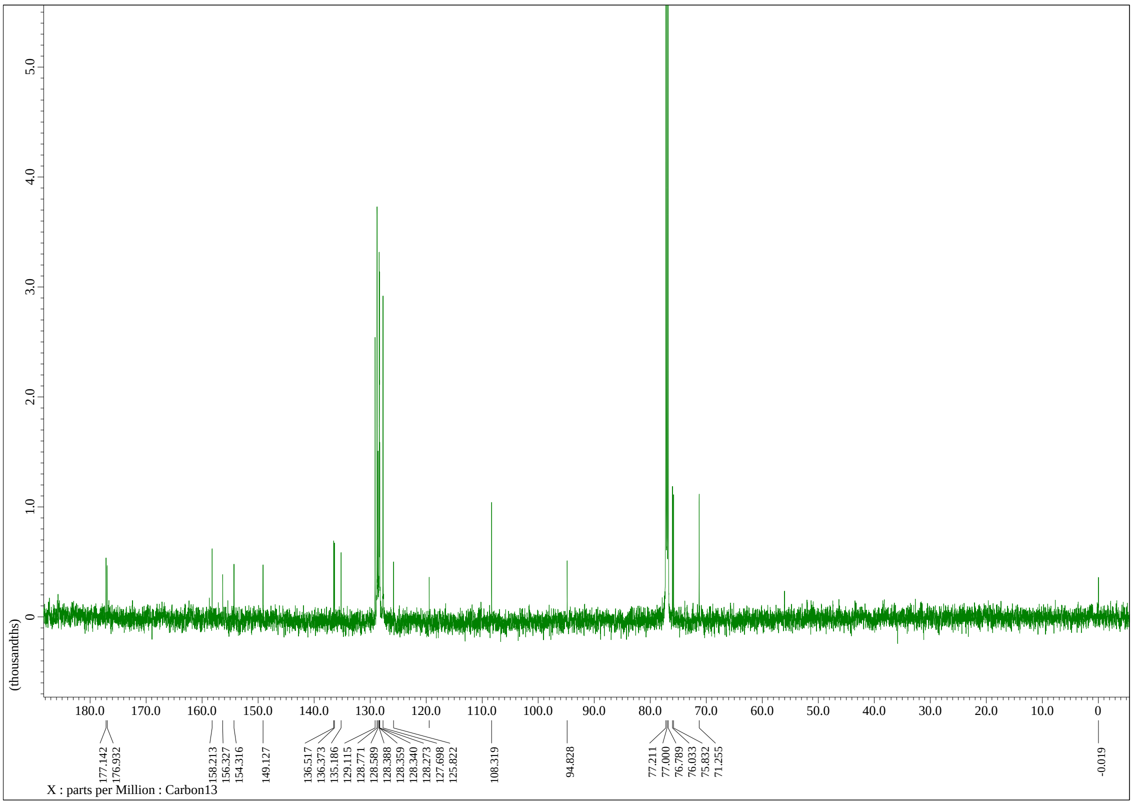

(thousandths)
177.142
176.932
158.213
156.327
154.316
149.127
136.517
136.373
135.186
129.115
128.771
128.589
128.388
128.359
128.340
128.273
127.698
125.822
108.319
94.828
77.211
77.000
76.789
76.033
75.832
71.255
-0.019
X : parts per Million : Carbon13

13

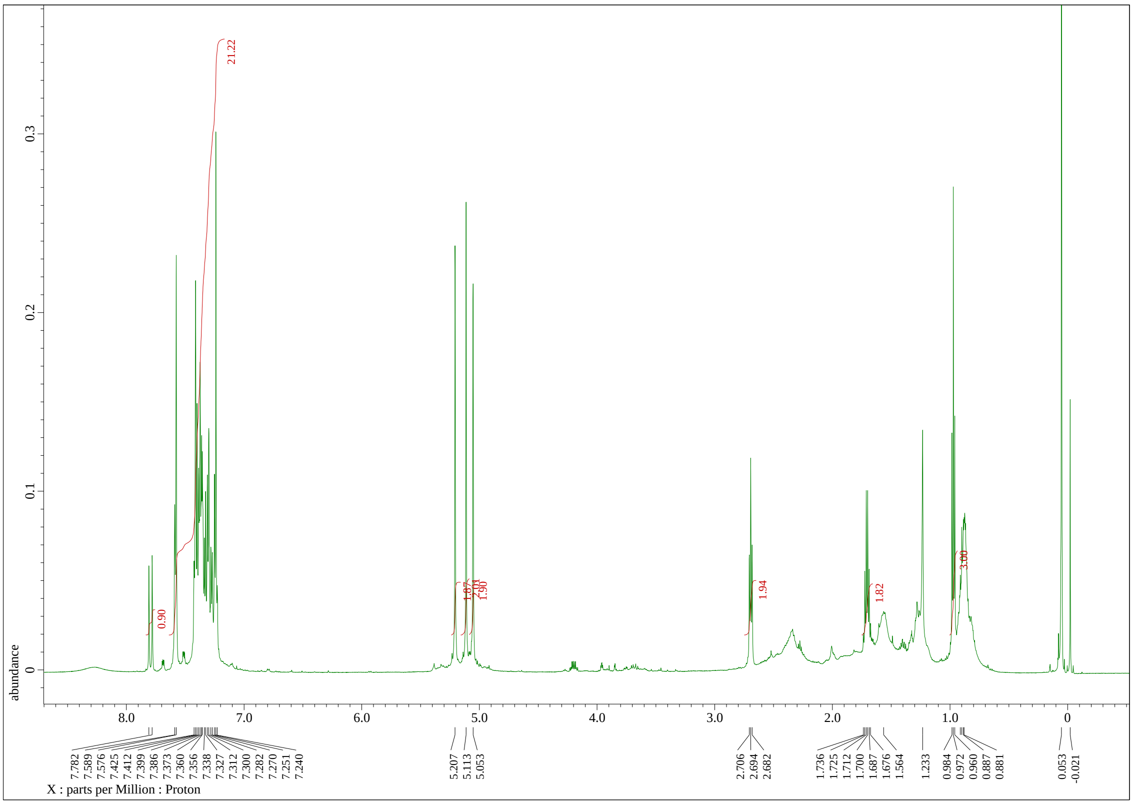

abundance
X : parts per Million : Proton

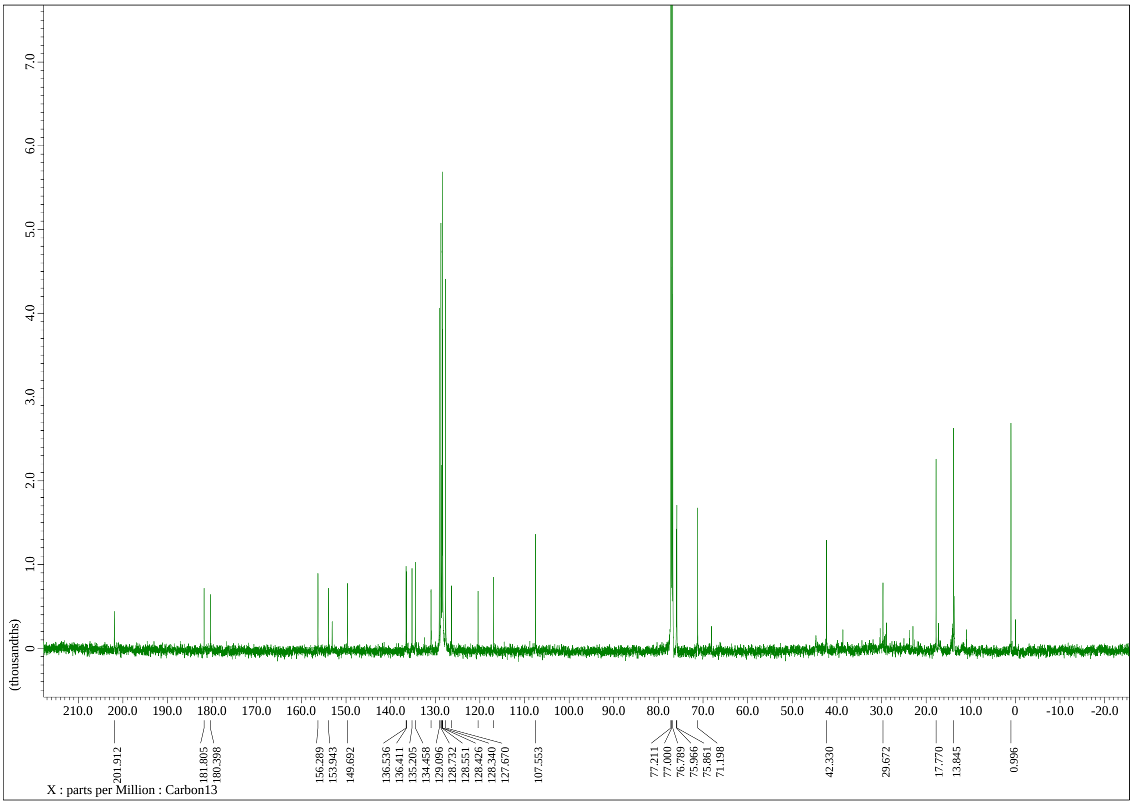

(thousandths)
X : parts per Million : Carbon13

1

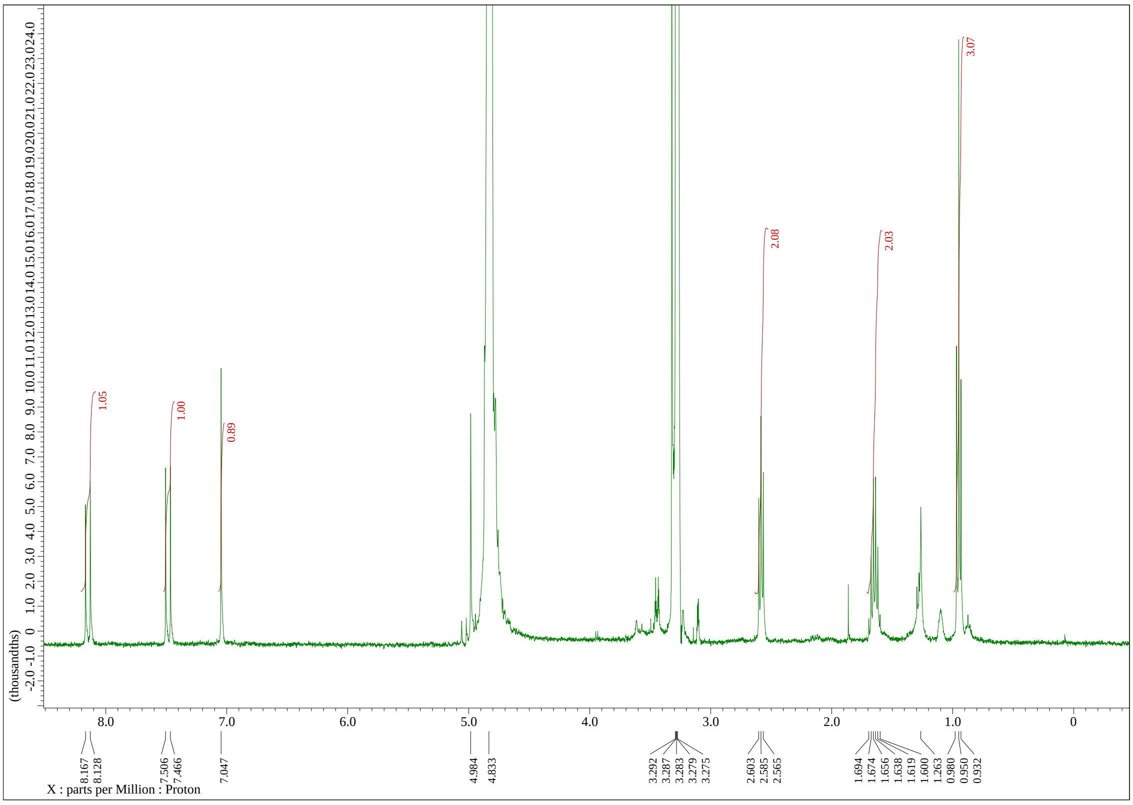

(thousandths)
X : parts per Million : Proton
8.167
8.128
7.506
7.466
7.047
4.984
4.833
3.292
3.287
3.283
3.279
3.275
2.603
2.585
2.565
1.694
1.674
1.656
1.638
1.619
1.600
1.263
0.980
0.950
0.932
1.05
1.00
0.89
2.08
2.03
3.07

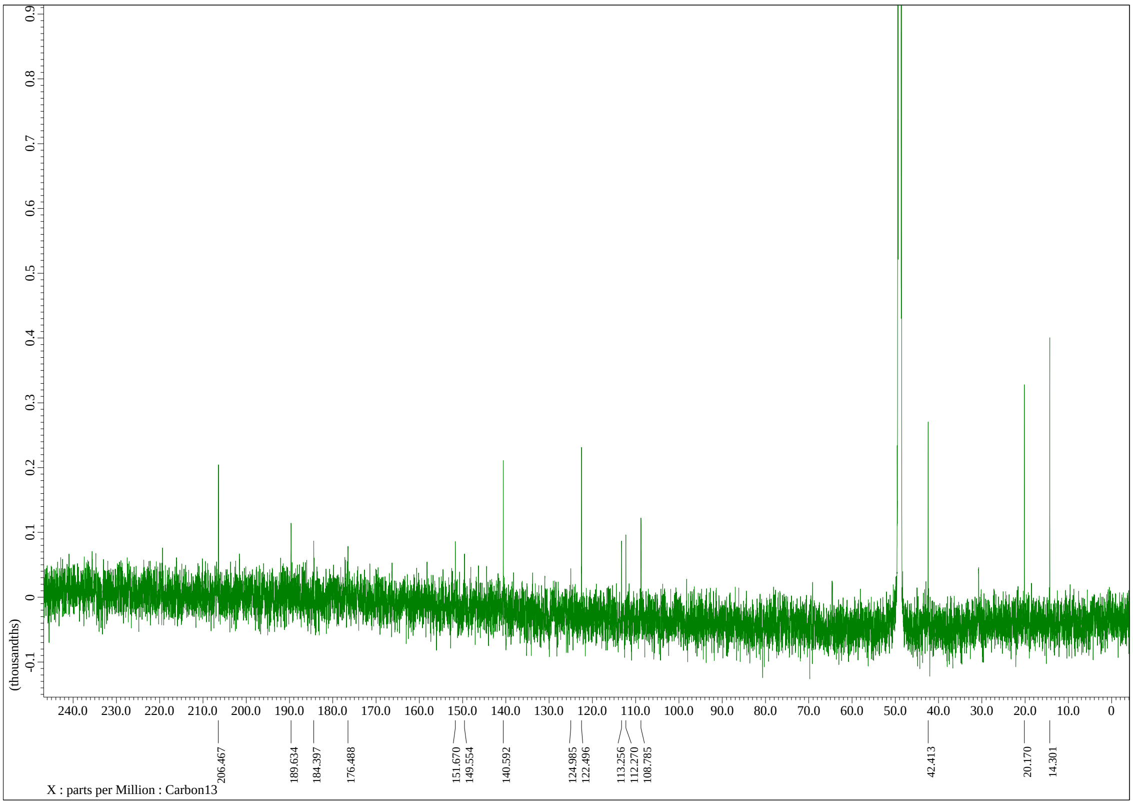

(thousandths)
X : parts per Million : Carbon13
206.467
189.634
184.397
176.488
151.670
149.554
140.592
124.985
122.496
113.256
112.270
108.785
42.413
20.170
14.301

15

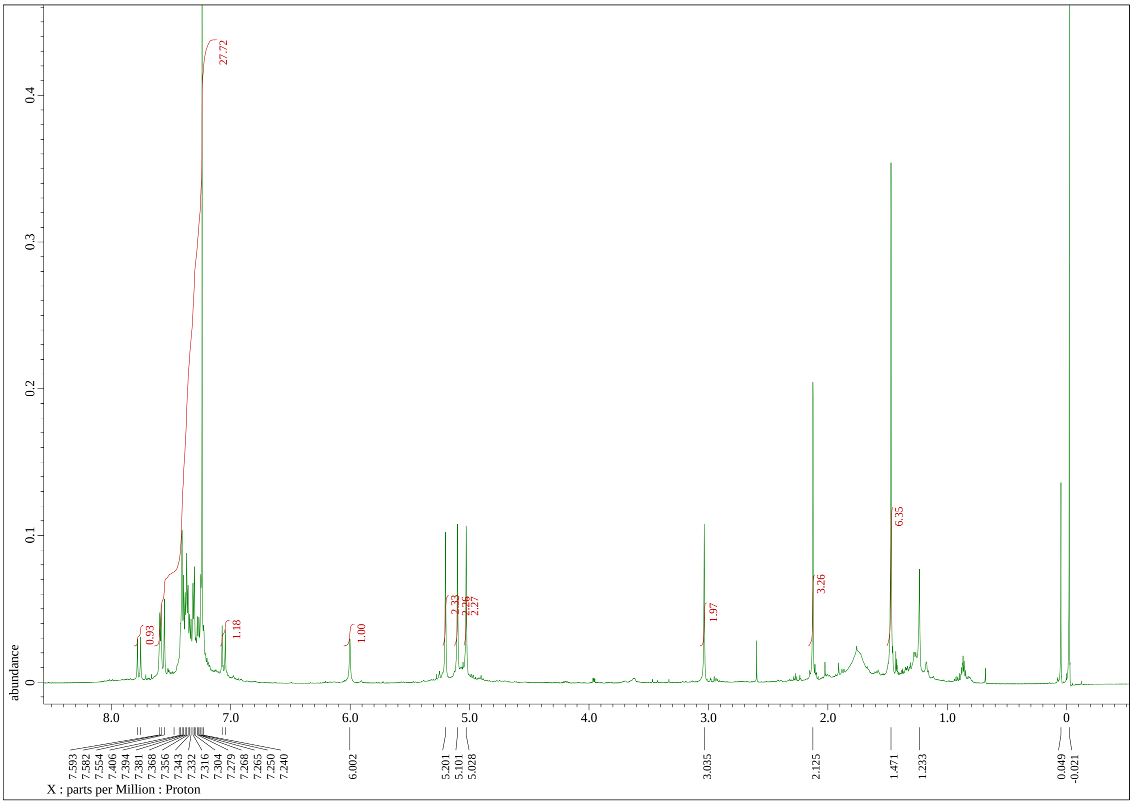

27.72
0.93
1.18
1.00
2.33
2.26
2.27
1.97
3.26
6.35
abundance
0.4
0.3
0.2
0.1
0
8.0
7.0
6.0
5.0
4.0
3.0
2.0
1.0
0
7.593
7.582
7.554
7.406
7.394
7.381
7.368
7.356
7.343
7.332
7.316
7.304
7.279
7.268
7.265
7.250
7.240
6.002
5.201
5.101
5.028
3.035
2.125
1.471
1.233
0.049
-0.021
X : parts per Million : Proton

14

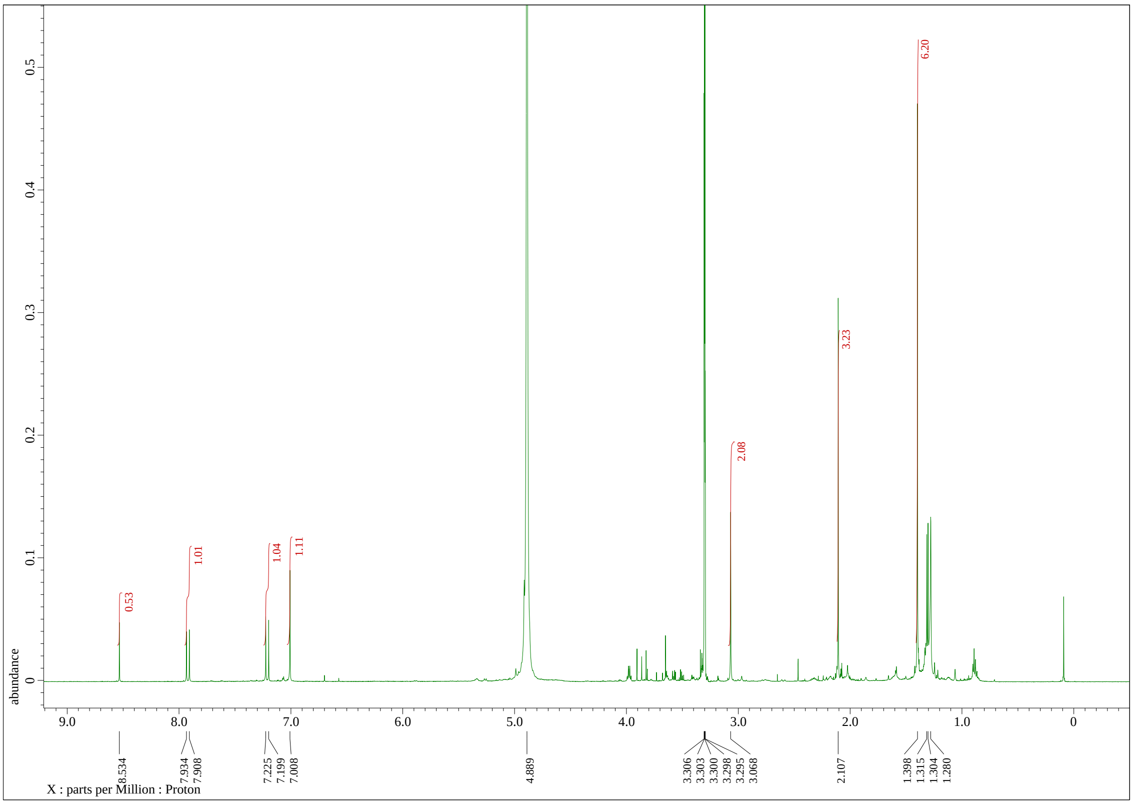

abundance
0.53
1.01
1.04
1.11
2.08
3.23
6.20
8.534
7.934
7.908
7.225
7.199
7.008
4.889
3.306
3.303
3.300
3.298
3.295
3.068
2.107
1.398
1.315
1.304
1.280
X : parts per Million : Proton

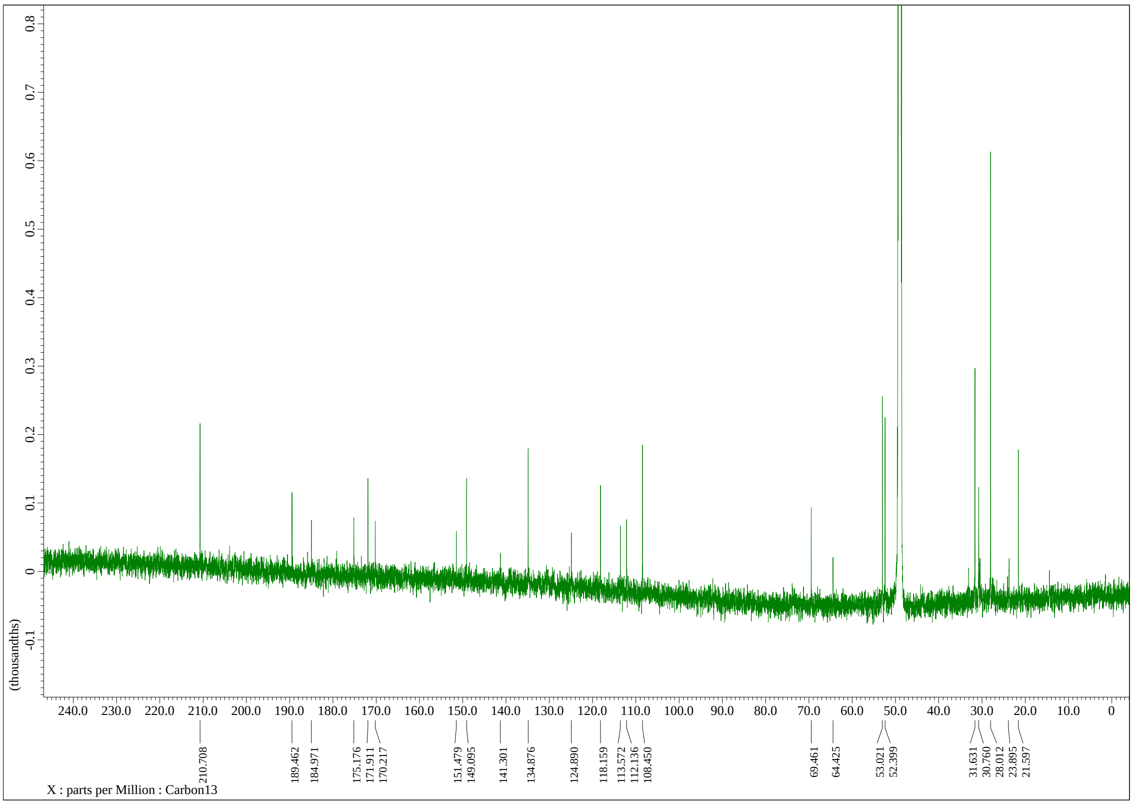

(thousandths)
210.708
189.462
184.971
175.176
171.911
170.217
151.479
149.095
141.301
134.876
124.890
118.159
113.572
112.136
108.450
69.461
64.425
53.021
52.399
31.631
30.760
28.012
23.895
21.597
X : parts per Million : Carbon13
